# The pivotal role of sleep in mediating the effects of life stressors and healthy habits on allostatic load in mid-life adults

**DOI:** 10.3389/fnhum.2024.1509223

**Published:** 2024-12-20

**Authors:** Ingrid Buller-Peralta, Sarah Gregory, Audrey Low, Maria-Eleni Dounavi, Katie Bridgeman, Georgios Ntailianis, Brian Lawlor, Lorina Naci, Ivan Koychev, Paresh Malhotra, John T. O'Brien, Craig W. Ritchie, Yves Dauvilliers, Graciela Muniz-Terrera

**Affiliations:** ^1^Edinburgh Dementia Prevention, Centre for Clinical Brain Sciences, Outpatients Department Level 2 Western General Hospital, University of Edinburgh, Edinburgh, United Kingdom; ^2^Scottish Brain Sciences, Edinburgh, United Kingdom; ^3^Department of Psychiatry, School of Clinical Medicine, University of Cambridge, Cambridge, United Kingdom; ^4^Trinity College Institute of Neuroscience, School of Psychology, Trinity College Dublin, Dublin, Ireland; ^5^Global Brain Health Institute, Trinity College Dublin, Dublin, Ireland; ^6^Department of Psychiatry, Oxford University, Warneford Hospital, Oxford, United Kingdom; ^7^Department of Brain Sciences, Imperial College London, London, United Kingdom; ^8^Mackenzie Institute, University of St Andrews, St Andrews, United Kingdom; ^9^Institute for Neurosciences of Montpellier, University of Montpellier, CHU Montpellier INSERM, Montpellier, France; ^10^Ohio University Heritage College of Osteopathic Medicine, Athens, OH, United States

**Keywords:** allostatic load, sleep quality, cognitive activities, healthy diet, sport habits, traumatic stressors, psychosocial stressors, resilience

## Abstract

**Objectives:**

We assessed the modulation of allostatic load (AL) by engagement in healthy habits and life stressors, mediated through resilience and the perceived influence of the stressors. Sleep was included as third mediator given extensive evidence associating to all the analysed factors.

**Methods:**

Structural equation models to assess the modulation of AL by either traumatic or psychosocial stressors and healthy habits were generated with data from 620 mid-life adults (age 51.3 ± 5.48 years). Model 1 included self-reported life stressors, engagement in cognitive and physical activities, resilience and a pyramid score for diet. In Model 2, self-reported sleep quality was included in the mediation analysis between resilience and perceived stress on AL.

**Results:**

Direct effects of sports and diet on AL, and on resilience by sports were found in all the evaluated models. The modulation of AL by both types of stressors was only revealed in model 2, through indirect effects of perceived influence via sleep quality. An effect of sport habits on AL via resilience was found to be mediated by sleep, and equivalent but opposed effects of perceived influence of stressors and resilience on sleep quality emerged as critical factor for AL modulation.

**Conclusion:**

Our results suggest that sleep plays a pivotal role in the modulation of AL by both life stressors and sport habits, balancing the harmful and protective effects of perceived stress and resilience. The relative weight of one over the other to worsen or improve sleep quality will determine the resulting level of AL.

## 1 Introduction

Allostatic Load (AL) describes the wear and tear resulting from physiological responses upon chronic stress exposure (McEwen, 1998; Schulkin, 2002). As an index composed of inflammatory, metabolic, and cardiovascular biomarkers (Duong et al., 2017; Buller-Peralta et al., 2024), AL summarises a global level of systemic dysfunction and has been associated with an increased risk of developing several pathological conditions including major depression, frailty, changes in brain volume, cardiovascular disease, chronic fatigue syndrome, fibromyalgia, diabetes type 2, seizures, breast cancer, cognitive impairment, and Alzheimer’s disease (AD) (Booth et al., 2015; Zsoldos et al., 2018; D’Amico et al., 2020; Guidi et al., 2021; Twait et al., 2023; Buller-Peralta et al., 2024). Various studies investigated factors associated with AL, from psychological to sociodemographic but in general, high levels of AL can result from three situations (Guidi et al., 2021). The first, and most known, is the repeated exposure to stressors that leads to a chronic enhancement of physiological arousal. This situation refers to both the number and frequency of stressors experienced through life. The second situation refers to the inability to successfully adapt to adversity and stress demands through effective coping strategies. These are reflected in the concept of resilience and can be considered as protective factors against allostatic overload (McEwen, 2008; Rösner et al., 2023). A third situation occurs when the stress response cannot be terminated after the end of the stressor exposure. Here, the physiological arousal triggered by fear or danger is unable to be extinguished and is usually re-experienced when exposed to cues or contexts related to the event, as seen in patients and rodent models of post-traumatic stress disorder (PTSD) (Jovanovic et al., 2012; Knox et al., 2012; Garfinkel et al., 2014; Lissek and van Meurs, 2015). Thus, an event that occurred a long time ago can still exert its arousing influence many years later to cause increased AL levels.

Besides resilience, lifestyle and habits have also been studied to identify protective factors against allostatic overload (Forrester et al., 2019). Among them, healthy dietary habits have been consistently associated with lower AL in both young and older adults (Mattei et al., 2013; Beydoun et al., 2019; Obomsawin et al., 2022; Zhou et al., 2022). Particularly, adherence to a Mediterranean diet has been frequently related to lower cardiovascular burden (Tong et al., 2016; Salas-Salvadó et al., 2018; Artegoitia et al., 2021; Gregory et al., 2023), which is a strong component of the AL index (Buller-Peralta et al., 2024). Similarly, regular engagement in physical activity has been associated with lower levels of AL in European, Latin-American and Asian populations (Gay et al., 2015; Upchurch et al., 2015; Petrovic et al., 2016; Zhang et al., 2022), with an effect not restricted to vigorous sports but also to mild and moderate occupational and leisure activities (Forrester et al., 2019; Bu and Li, 2023). Cognitive habits, such as reading, practicing another language, painting, or playing an instrument, have also been found to be protective against stress exposure (García-Moreno et al., 2021) and associated with lower levels of AL (Wang et al., 2021). In addition, sleep has received special attention in relation to AL and stress exposure. While poor sleep quality has been found to be associated with higher levels of AL (McEwen, 2006; Clark et al., 2014; Bei et al., 2017; Hux et al., 2017; Christensen et al., 2022), evidence also reports impaired sleep upon stress exposure (Jean Kant et al., 1995; Van Reeth et al., 2000; Kim and Dimsdale, 2007; Mellman et al., 2007; Pawlyk et al., 2008; Ackermann et al., 2019) and has been identified as a key symptom of PTSD (Lancel et al., 2021).

In the present study, we analysed how lifetime stressors, and a healthy lifestyle indicated by adherence to a healthy diet cognitive engagement and physical activity modulate AL in a British cohort of cognitively normal mid-life adults. By including the self-reported perceived influence of the stressor events and the levels of resilience as potential mediators, we evaluated if the modulation depends also on the ability to cope and overcome the allostatic demands. Following the extensive evidence of the bidirectional relation between stress and sleep quality, we further assessed its role in the modulating effects of AL.

## 2 Materials and methods

### 2.1 Participants

Data from the PREVENT study [v700 baseline dataset (Ritchie et al., 2024)] were used. As described previously (Ritchie and Ritchie, 2012; Ritchie et al., 2013), the PREVENT cohort recruited mid-life participants (age: 40–59 years) from sites in Edinburgh, West London, Dublin, Cambridge and Oxford. All participants provided written informed consent before participation and were free of cognitive impairment at the baseline visit.

### 2.2 Ethics

Multi-site ethical approval was granted by the UK London-Camberwell St Giles National Health Service (NHS) Research Ethics Committee (REC reference: 12/LO/1023, IRAS project ID: 88938), which operates according to the Helsinki Declaration of 1975 (and as revised in 1983). Separate ethical approval was received for the Dublin site, from Trinity College Dublin School of Psychology Research Ethics Committee (SPREC022021-010) and the St James Hospital/Tallaght University Hospital Joint Research Ethics Committee. All substantial protocol amendments have been reviewed by the same ethics committees with a favourable opinion granted before implementation at sites.

All necessary participant consent has been obtained before assessments and the appropriate institutional forms have been archived. Any patient/participant/sample identifiers included were not known to anyone (e.g., hospital staff, patients, or participants themselves) outside the research group so cannot be used to identify individuals. Participants in the study remained anonymous, identifiable information was held at site and only accessible by the direct research team. This identifiable information was not shared to the study data base where each participant was only identified by a study ID number.

### 2.3 AL scoring

#### 2.3.1 Biomarkers collection

Blood samples were collected in a fasted state during the baseline visit and analysed immediately at local laboratories analysed in local laboratories for biochemistry and haematology measures using NHS standard procedures (Ritchie et al., 2024). Vital signs were collected after breakfast by trained members of the research team. Blood pressure and heart rate were collected in triplicate (both supine and standing) and a mean of the three measures. Height and weight were recorded for body mass index (BMI) calculation and measurements of waist and hip circumference were documented. Fourteen biomarkers were assessed for inflammatory/immune (creatinine, albumin, C-reactive protein [CRP], fibrinogen), cardiovascular (systolic blood pressure [SBP], diastolic blood pressure [DBP], resting heart rate [RHR], and waist-to-hip ratio [WHR]), and metabolic (total cholesterol, high-density-lipoprotein [HDL] cholesterol, low-density-lipoprotein [LDL] cholesterol, glycemia, triglycerides, and BMI) systems.

#### 2.3.2 Comprehensive AL score (ALCS)

All biomarkers were scored to create a comprehensive AL index (ALCS), as previously described (Buller-Peralta et al., 2024). Initial categories for “no-risk” (zero points), “at-risk” (one point), and “high-risk” (two points) were defined for each biomarker, based on both clinical thresholds (National Institute for Health and Clinical Excellence (NICE), 2006; Fuggle, 2018) and quartiles from sex-specific distributions. When the clinical upper limit (clinical-up) was higher than the 75th percentile (p75: creatinine, triglycerides, CRP, SBP, DBP), the at-risk category was defined between ≥p75 – ≤clinical-up (no-risk: <p75 and high-risk: >clinical-up). When the clinical upper limit was lower than p75 (total cholesterol, LDL cholesterol, BMI, WHR), at-risk was defined between ≥clinical-up – ≤p75 (no-risk: <clinical-up, high-risk: >p75). For reverse biomarkers (albumin, HDL cholesterol), if the clinical lower limit (clinical-low) was below the 25th percentile (p25), at-risk was defined as ≤ clinical-low – ≥p25 (no-risk: >p25 and high-risk: <clinical-low). For RHR, only clinical categories provided by the British Cardiovascular Society for age and sex were used.

Medication treatments coded through the Anatomic Therapeutic Chemical (ATC) classification system (Nahler, 2009) were scored as high-risk to account for potential masking some biomarkers values, as follows: total cholesterol, triglycerides and LDL for lipid modifying agents (C10); systolic and diastolic blood pressure for anti-hypertensive medication (C02, C03, C09); resting heart rate for beta-blockers (C07) or calcium blockers (C08); and glycemia for insulin or analogues (A10).

The summed scores were used as continuous variable for the mediation analysis. The decision algorithm is described in Supplementary Figure S1, and clinical thresholds and quartiles values are detailed in Supplementary Table S1.

### 2.4 Life stressors

Stressful life events were assessed through the self-reported Life Stressor Checklist—Revised (LSC-R) (Wolfe et al., 1997), where participants are asked if they had experienced a set of 30 stressors across their lives, such as natural disasters, sexual assault, death of a relative, divorce, etc. Participants also report their age at the time of the event, age when the event ended, belief that they were in harm (“yes” or “no”), feelings of helplessness (“yes” or “no”), and the perceived influence of the event over the past year (rated on a five-point intensity scale from 1 = “not at all or never” to 5 = “extremely”) (Wolfe et al., 1997). Items 29 and 30 are open questions to identify other stressors not listed before, so they were excluded from the current analysis as many answers were found to replicate the same events listed previously, leaving a maximum total score of 28 possible life stressors. Traumatic events were identified following the definition proposed by the Diagnostic and Statistical Manual of Mental Disorders (DSM-5) criteria A for post-traumatic stress disorder (PTSD) diagnosis, as an exposure by direct experience, witnessing or learning, to actual or threatened death, serious injury, or sexual violence (American Psychiatric Association, 2013). Similarly, the International Classification of Diseases 11th Revision (ICD-11) defines stressful events as an extremely threatening situation, including “*natural or human-made disasters, combat, serious accidents, torture, sexual violence, terrorism, assault.… witnessing the threatened or actual injury or death of others in a sudden, unexpected, or violent manner; and learning about the sudden, unexpected or violent death of a loved one*” (World Health Organization, 2022). In these proposals, events not involving an immediate threat to life or physical injury such as divorce or job loss are considered psychosocial stressors, and medical incidents involving natural causes, such as a heart attack, are not considered traumatic (Pai et al., 2017). Thus, items 1–3, 12, 13, 17, 19–23, 25–28 were classified as traumatic stressors, and items 4–11, 14–16, 18, 24 were classified as psychosocial stressors (description of items in Supplementary material S1).

### 2.5 Healthy habits

#### 2.5.1 Pyramid score

The Pyramid score is a widely used scoring algorithm to evaluate adherence to a Mediterranean-style diet (Bach-Faig et al., 2011). Each contributing food component was coded on a continuous scale of 0–1 with a total possible score of 15 points and was calculated as previously described (Tong et al., 2016; Gregory et al., 2023). Briefly, scores were derived from the Scottish Collaborative Group Food Frequency Questionnaire (SCG-FFQ), which gathered data on 175 different foods and drinks consumed by participants over the last two to three months. Total energy intake (kcal/day) was derived from the dataset and included in the analysis. Participants with extreme energy intakes (<600 kcal, >6,000 kcal) were excluded from the analysis (for full details see Supplementary Table S2).

From the 620 participants selected with complete data for AL scoring, 570 had sufficient data to calculate a Pyramid score. To be able to obtain modification indices for structural equation models (SEMs) and estimate indirect effects via bootstrapping, missing values for the remaining 50 participants were imputed by a regression imputation.

#### 2.5.2 Cognitive and sports habits

Healthy habits related to physical or cognitive activities were evaluated through selected items from the Lifetime of Experiences Questionnaire (LEQ) (Valenzuela and Sachdev, 2007). Participants are asked to report on average, how often they take part in each activity (rated on a six-point intensity scale from 0 = “never” to 5 = “daily”) from the age of 30 until the end of their working life or present (if still in work). Cognitive habits were calculated by summing the scores for items related to the play/practice a musical instrument, artistic past-time, reading and practicing a second language, whereas sports habits summarised scores related to the practice of mild, moderate, or vigorous physical activities. Full item descriptions are detailed in Supplementary material S2.

### 2.6 Mediators

#### 2.6.1 Perceived influence of stressors over the past year

To discriminate between the effects derived from the number of life stressors and their actual impact at the time of evaluation, scores of the self-perceived influence over the past year obtained by the LSC-R were derived separately for traumatic and psychosocial stressors, and included as a mediator between the exogenous variables and AL.

#### 2.6.2 Resilience

As a potential protective factor mediating the effect of stressors on AL, an index of self-reported resilient attitudes was included through the Connor-Davidson Resilience Scale (CDRS) (Connor and Davidson, 2003). This provides an overall index for self-reported resilient attitudes through a 25-item Likert scale, with points ranging from 0 to 4, and higher scores reflecting greater resilience.

#### 2.6.3 Poor sleep quality

Self-reported sleep quality was assessed through the Pittsburgh Sleep Quality Index (PSQI) questionnaire (Buysse et al., 1989). It summarises seven components of sleep quality (self-perceived quality, latency, duration, efficiency, disturbance, medications, and daytime dysfunction) in a total score ranging between 0 and 21, with higher scores reflecting poorer sleep quality.

### 2.7 Demographic covariates

Demographic covariates included age, sex (categorical, coded as males = 1, females = 2), and years of education.

### 2.8 Structural equation models for assessing mediation path analysis

Structural equation models (SEMs) path analysis is a generalization of multiple regression procedures that allows to include multiple endogenous (dependent) variables, to impose restrictions on one or more parameters (i.e., set a parameter to zero, set two parameters to be equal, or restrict the assessment of unnecessary regressions), and to assess indirect effects through chained associations between an exogenous (independent) variable and mediating endogenous variables (Kline, 2011; Byrne, 2016b). The latter is crucial when no direct associations between a predictor and an outcome are found but can be theoretically hypothesised to be mediated indirectly through an additional association with another variable between them.

**Figure 1:**
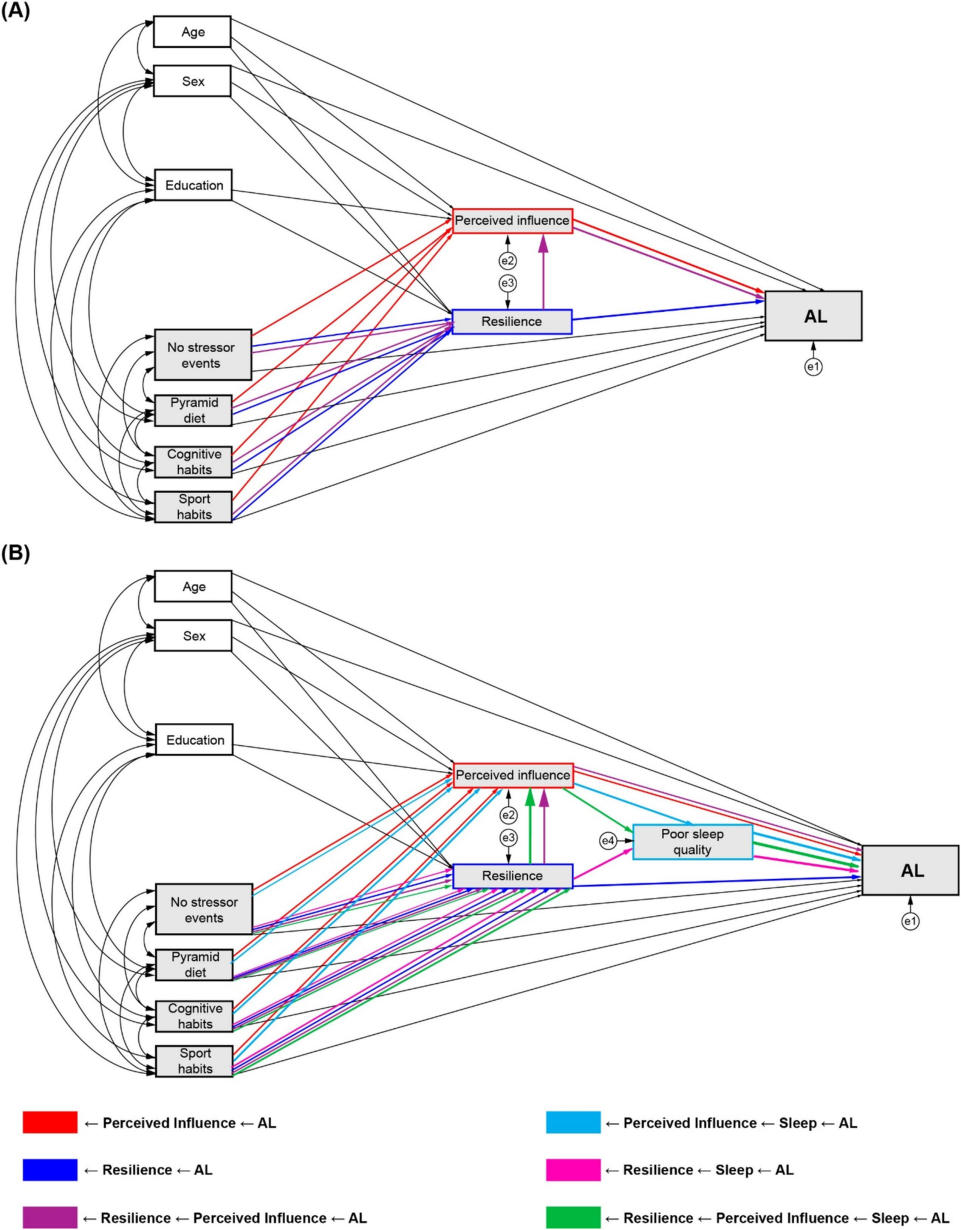
Conceptual mediation models and analysed indirect pathways. **(A)** Model 1 with perceived influence and resilience as mediators. **(B)** Model 2 with poor sleep quality, perceived influence, and resilience as mediators.

Based on the reviewed evidence, we developed a conceptual framework for SEM path analyses with the following exogenous variables: *(i) Experiential factors*, including the number of stressors (traumatic or psychosocial), Pyramid score, cognitive habits, and sport habits; and *(ii) Demographic factors*, including sex, age, and years of education covariates. In the first model (Model 1), we evaluated three *a priori* potential mediating pathways that may play a role in the associations between the influencing factors and AL: *(i)* through the perceived influence of the stressors; *(ii)* through resilience; and *(iii)* through a chain mediation of resilience on the perceived influence (full details in Figure 1A).

Following the results of the first model, and according to the theoretical evidence reviewed, sleep quality was included as a third mediator between the first two (perceived influence and resilience) and AL (Model 2), adding three new potential mediating pathways to the previously described: *(iv)* sequentially through perceived influence to sleep; *(v)* through resilience to sleep; and *(vi)* through resilience to perceived influence to sleep (full details in Figure 1B). No direct effects paths from traumatic or psychosocial stressors to poor sleep quality were specified, as evidence reports that the effects of stressor exposure on sleep does not follow a uniform dose–response relationship but can vary between individuals, and additional factors such as stressor chronicity, resilience ability and the event appraisal need to be accounted to estimate the impact of differential sleep impact in response to stress (Kalmbach et al., 2018). Therefore, we hypothesised than the mediating effect of sleep between the exogenous variables and AL had to arise from a previous indirect effect via resilience, perceived influence or resilience to perceived influence.

### 2.9 Statistical analysis

All analyses were conducted using IBM SPSS Statistics v.27.0 (IBM Corp., 2020) and Amos (Analysis of Moment Structures) v.27.0 (Arbuckle, 2020). Statistical differences between sex for all demographic and behavioural variables were evaluated by two-sided Mann Whitney-U rank sum tests, as normality estimated by Shapiro–Wilk tests were below the rejection value of <0.05 (details in Supplementary Table S3).

Pearson’s product moment correlation coefficients were computed for preliminary evaluation of bivariate associations among all the studied variables.

#### 2.9.1 Model identification

In order to conduct SEM path analysis, identification of the hypothesised path models was checked through the compliance with the T-rule (necessary but not sufficient) and the recursive rule (sufficient but not necessary) (Kline, 2011; Byrne, 2016b).

The T-rule states that for a model to be sufficiently identified, the number of known parameters (calculated as k*(*k* + 1)/2 + *k*, where *k* is the number of observed exogenous and endogenous variables) must be equal to (just-identified model) or greater than (over-identified model) the number of free parameters that needs to be estimated. All the evaluated models complied with the rule for over-identification (Model 1, Traumatic stressors: known = 65 > estimated = 58; Model 1, Psychosocial Stressors: known = 65 > estimated = 59; Model 2, Traumatic stressors: known = 77 > estimated = 63; Model 2, Psychosocial Stressors: known = 77 > estimated = 63).

Thus, all assessed models were over-identified and recursive, given no correlated residuals, bi-directional effects, or feedback loops were included.

#### 2.9.2 Assessments of required assumptions for SEM analysis

##### 2.9.2.1 Normality assumption

As shown by the use of Mann Whitney-U rank sum tests for males vs. females comparisons (Supplementary Table S3) all assessed variables showed deviations from normality (assessed by Shapiro–Wilk test). However, for sample sizes greater than 300, either an absolute skew value larger than 3 or an absolute kurtosis (proper) larger than 8 may be used as reference values for determining substantial non-normality (West et al., 1995; Kline, 2011). Both skewness and kurtosis values for each variable were assessed and reported in Supplementary Table S4, with only influence of traumatic events showing a significant deviation from kurtosis normality (kurtosis = 8.89). However, all multivariate normality Mardia’s coefficients calculated for each SEM model showed significant deviations from normality (details in Supplementary Table S4). For the purpose of SEM analysis, only normality for the endogenous variables (AL, sleep quality, resilience and perceived influence) is required for accurate estimation of Maximum Likelihood, and violations of normality can both inflate likelihood-ratio *χ*^2^ tests—leading models to be rejected more often than they should—and underestimate standard errors—increasing the probability of error type I when testing significance of individual parameters (Byrne, 2016b). To address this violation, a bootstrapping approach was used, as one of the most recommended for samples large enough to be representative where univariate and multivariate normality assumptions are not met (Hancock and Liu, 2012; Byrne, 2016a; Johnston and Faulkner, 2021). The bootstrapping procedure consists in generating multiple new samples—at least 1,000 (Streukens and Leroi-Werelds, 2016)—from the original database, to construct a bootstrap sampling distribution that will operate in a similar way as those traditionally associated with parametric inferential statistics (i.e., t or F distributions), but without the need to meet the normality assumption for an adequate estimation of parametric values, such as regression coefficients (Hancock and Liu, 2012; Byrne, 2016a). As this sample-based distribution is drawn through consecutive replacements of the original sample, it allows to generate bias-corrected standard errors and confidence intervals for an accurate estimation of statistical significance, correcting the increased probability of error type-I caused by the violation of normality. Therefore, a 1,000-samples bootstrapping was performed for all the SEM models analysed, with bias-corrected confidence intervals (CI) set at 95%. Only bias-corrected standard errors, CI, and adjusted *p*-values are reported for direct and indirect effects.

##### 2.9.2.2 Linearity assumption and collinearity assessments

Linearity assumption was checked on each pair of associations between dependent and independent variables analysed in the models. Deviation from linearity is calculated on SPSS as follows: after subtracting the within groups form residuals sum of squares, difference is divided by degrees of freedom (df) of residuals—df within group to the deviation mean squared. An *F* value is computed as the mean square ratio of deviation/within groups and the *p* value is calculated with the corresponding degrees of freedom (df deviation, df withing groups). *p*-values <0.05 indicate a significant deviation from linearity. No violations of linearity were found on any relation between dependent and independent variables assessed by the models, as shown in Supplementary Table S5.

The presence of multicollinearity was checked by performing a multiple regression analysis on each of the dependent variables assessed in the SEM models, with the corresponding independent variables as covariates. Variance inflation factor (VIF) >5 and tolerance statistic <0.2 were considered as indicators of multicollinearity. Results are summarised in Supplementary Table S5, showing no collinearity effects.

#### 2.9.3 Imputation of missing values of the Pyramid score variable

To obtain modification indexes, bias-corrected standard errors, CI, and adjusted p-values through bootstrapping procedures in the fitted SEM models, the full dataset is required to be complete. To choose the best procedure to deal with the 50 missing values of the Pyramid score variable, the missing data mechanism operating needed to be determined. Although there is no formal testing to assess the missing data mechanism that is present in the data, we performed a logistic regression to assess for the plausibility of either a missing at random (MAR) or missing completely at random (MAR) assumptions (Fielding et al., 2009). In brief, if significant relations between missing Pyramid Score data and all the rest of the variables in the dataset were found, then MAR could be assumed. If no relationships are shown, then MCAR can be concluded. The possibility of missing not-at random (MNAR) assumption was discarded, as it implies that missing data is systematically related to events or factors that were not measured in the study, which cannot be plausible when only an 8.1% of data is missing for one variable (Little and Rubin, 2002). Therefore, after transforming missing and non-missing Pyramid scores into dichotomic categories, where 0 corresponded to non-missing values and 1 corresponded to missing values, a logistic regression on the full dataset was performed setting non-missing (values = 0) as reference category. Given no significant associations were found between the missing data and any the variables (see full report in Supplementary Table S6), we concluded that it was possible to assume that missing data were MAR, and hence, assumptions for performing imputation were satisfied.

Robust Full Information Maximum Likelihood (RFIML) imputation method was then performed on AMOS, as suggested by previous research to obtain better results with MCAR data and to allow for bias-correction to deal with non-normal data (Savalei and Falk, 2014; Jia and Wu, 2022). In brief, after fitting the model using the full information to calculate maximum likelihood, model parameters are set equal to their maximum likelihood estimates and linear regression is used to predict the unobserved values for each case as a linear combination of the observed values for that same case (Arbuckle, 2020). The advantage of using this method for complex SEM path analysis is that it considers all the relations assessed in the model instead of relying only on one particular set of associations with the variable to be predicted (Byrne, 2016b).

#### 2.9.4 Assessments of goodness of fit and modification indices

Hypothesised structural models were evaluated for goodness of fit by the following indices and criteria (Schermelleh-Engel et al., 2003; Schreiber, 2008): Likelihood-ratio *χ*^2^ test (*p* ≥ 0.05), Comparative Fit Index (CFI ≥ 0.95) and Tucker-Lewis Index (TLI ≥ 0.95), the root-mean-square error of approximation (RMSEA<0.06), and the standardised root mean squared residual (SRMR<0.08). If an initial model assessment showed poor fit for the *χ*^2^ test (*p* < 0.05), calculated modification indices (MI) were considered for post-hoc model modifications. The suggested parameters to add for model improvement were selected from MI values greater than 3.84 as indication of significant improvement (Harrington, 2008), and if they were consistent with the theoretical construct.

Direct effects estimates were estimated as standard regression coefficients, whereas total effects were estimated but not considered for analysis due to the multiple pathways and mediations involved might lead to confounding interpretations.

For evaluation of total indirect effects of the mediating pathways of interest (see above and Figure 1), products of the standardised coefficients from the sequential mediation paths were labelled and set as user-defined estimands on AMOS. standardised estimates and *p*-values were calculated by bootstrapping, with 1,000 bootstrap samples, and bias-corrected percentile method, with confidence intervals at 95%. All significant effects were set at *α* = 0.05.

## 3 Results

### 3.1 Demographic and behavioural characteristics

The analytical sample included 620 participants from the PREVENT dementia study (61.13% females), with an average age of 51.3 (SD = 5.48) years old (females: 50.97, SD = 5.41), and a mean of 16.62 (SD = 3.44) years of education. Demographic and behavioural characteristics of the PREVENT participants included in the analyses are detailed in Table 1. Comparisons between sexes revealed higher AL scores and greater engagement in sports in males. Females showed higher Pyramid scores and an increased number and perceived influence of psychosocial stressors. No differences were found between males and females for age, years of education, number and perceived influence of traumatic stressors, resilience, and sleep quality (details in Supplementary Table S3).

**Table 1:** Demographic and behavioural characteristics of the studied population.

Variable [missing]				
Sex, *n* (%)			Male	241 (38.87%)
			Female	379 (61.13%)
		Total	Males	Females
Age, mean (SD)	Total range 40–60 (IQR = 9)	51.26 (5.48)	51.71 ± 5.56	50.97 (5.41)
Years of education, mean (SD)		16.62 (3.44)	16.35 ± 3.2	16.79 (3.58)
Allostatic load score, mean (SD)		6.22, (3.93)	6.88 ± 3.99	5.79 (3.83)
Pyramid diet score, mean (SD) [50]		8.13 (1.52)	7.71 ± 1.43 [16]	8.4 (1.51) [34]
Cognitive habits, mean (SD)	Total	7.37 (3.00)	7.17 ± 3.12	7.5 (2.92)
*Frequency by activity type, mean (SD)*	*Play/practice musical instrument*	0.49 (1.17)	0.54 (1.25)	0.47 (1.11)
	*Practice artistic pastime*	1.04 (1.59)	0.8 (1.46)	1.19 (1.65)
	*Reading*	4.76 (0.77)	4.74 (0.87)	4.78 (0.69)
	*Speaking second language*	1.07 (1.5)	1.08 (1.5)	1.06 (1.50)
Sport habits, mean (SD)	Total	10.83 (2.85)	11.29 ± 2.76	10.54 (2.88)
*Frequency by activity type, mean (SD)*	*Mildly energetic*	4.55 (0.76)	4.51 (0.73)	4.57 (0.77)
	*Moderately energetic*	3.75 (1.12)	3.83 (1.11)	3.7 (1.12)
	*Vigorous*	2.53 (1.73)	2.94 (1.61)	2.27 (1.76)
No of stressor events mean (SD)	Traumatic stressors	2.49, (1.97)	2.54 ± 1.94	2.46 (1.99)
	Psychosocial stressors	2.33, (1.57)	2.53 ± 1.73	2.46 (1.63)
Perceived influence during past year, mean (SD)	Traumatic stressors	4.42, (4.71)	4.12 ± 4.65	4.61 (4.74)
	Psychosocial stressors	4.85, (4.55)	4.34 ± 4.16	5.18 (4.76)
Connor-Davidson Resilience Scale (CDRS)	Total score, Mean (SD)	72.83 (12.98)	73.61 ± 12.43	72.33 (13.31)
Pittsburgh Sleep Quality Index Questionnaire (PSQI)	PSQI global score, Mean (SD)	6.03 (3.07)	5.84 ± 3.02	6.16 (3.1)

SD, Standard deviation; %, percentage.

### 3.2 Bivariate correlations

**Figure 2:**
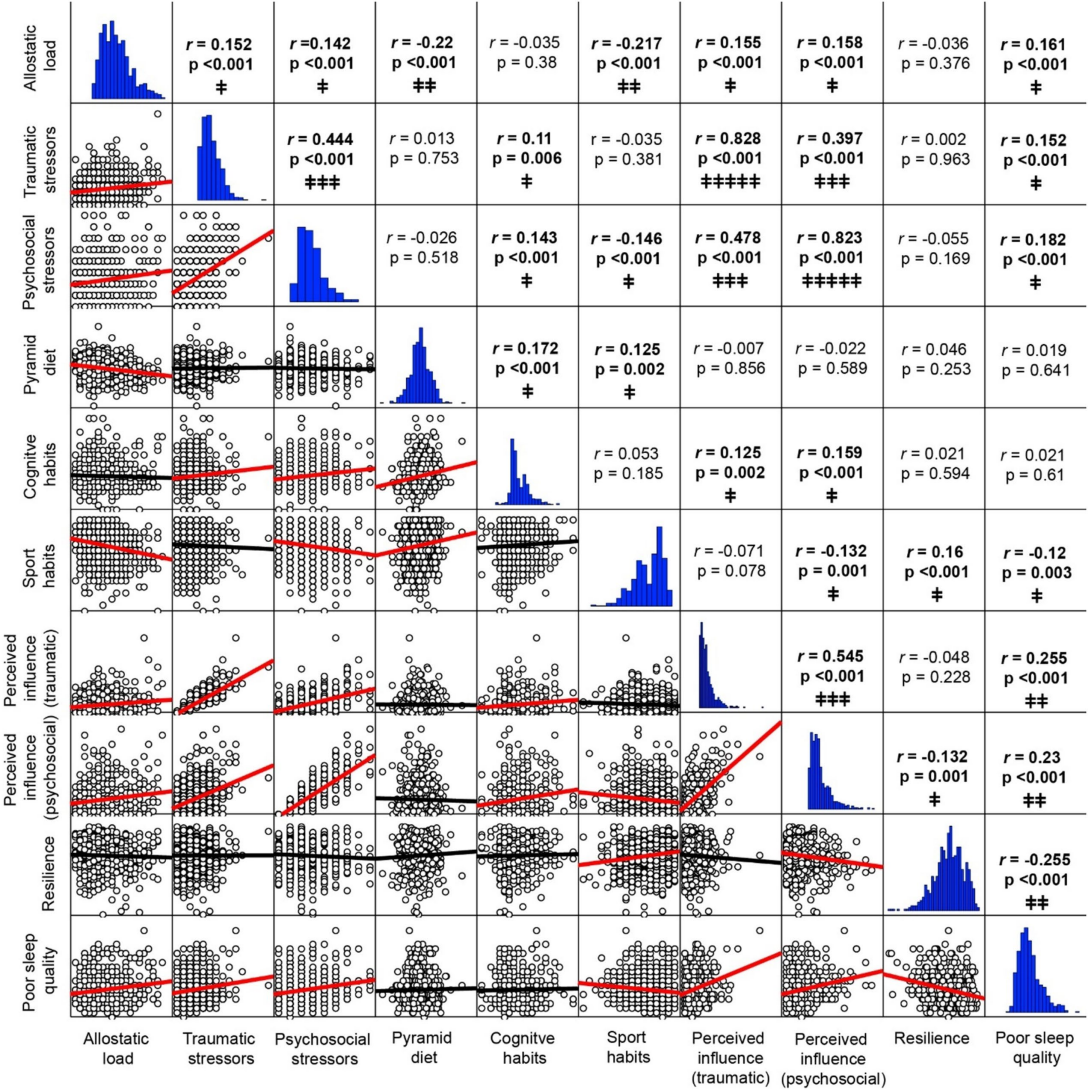
Bivariate correlations. *Upper triangle:* Pearson correlation coefficients and 2-tailed *p*-values (bold text denote significant *p*-values <0.05). *Lower triangle:* Correlation plots with lines of best fit (red lines denote significant correlations). *Horizontal graphs:* distribution histograms. *Correlations strength:* † very weak; ‡‡ weak; ‡‡‡ moderate; ‡‡‡‡ strong; ‡‡‡‡‡ very strong. For full correlations with demographic variables sex, age and years of education see Supplementary Table 7.

As shown in Figure 2 and Supplementary Table S7 detailing bivariate correlations results, small to moderate significant correlations were found between all variables assessed, except for two very strong correlations between number of stressors and their corresponding perceived influence scores (traumatic *r* = 0.828 and psychosocial *r* = 0.823, respectively). Among demographic covariates (see full details in Supplementary Table S7), small significant correlations were found in males between AL and sport habits, and in females between the Pyramid score, psychosocial stressors, and their perceived influence. Age showed a weak negative correlation to years of education and positive to higher AL, whereas higher education was weak but significantly correlated to lower AL, the Pyramid score and engagement in cognitive activities.

Significant but weak correlations were found between AL and the number of traumatic (*r* = 0.152) and psychosocial stressors (*r* = 0.142), their respective perceived influence (traumatic *r* = 0.155 and psychosocial *r* = 0.158), poor sleep quality (*r* = 0.161), smaller pyramid diet scores (*r* = −0.220) and sports habits (*r* = −0.217).

The number of traumatic stressors was weakly correlated with cognitive habits (*r* = 0.110) and poor sleep quality (*r* = 0.152) and had a moderate correlation to psychosocial stressors (*r* = 0.444) and their perceived influence (*r* = 0.397). Similarly, psychosocial stressors were moderately correlated to the perceived influence of traumatic stressors (*r* = 0.478), and weak but significantly correlated to cognitive habits (*r* = 0.143), poorer sleep quality (*r* = 0.182), and less engagement in sport habits (*r* = −0.146).

Higher Pyramid scores were correlated with both cognitive and sports habits (*r* = 0.172 and *r* = 0.125, respectively), whereas more frequent engagement in cognitive activities was correlated with a higher perceived influence of both traumatic (*r* = 0.125) and psychosocial stressors (*r* = 0.159). Sports habits showed weak but significant correlations to higher levels of resilience (*r* = 0.160), better sleep quality (*r* = 0.120) and lower perceived influence of psychosocial stressor (*r* = −0.132), while poor sleep quality was correlated with higher influence of traumatic (*r* = 0.255) and psychosocial stressors (*r* = 0.230), and lower resilience levels (*r* = −0.255).

### 3.3 Effects of life stressors and healthy habits on AL mediated by perceived influence and resilience

To assess the association of stressful life events and healthy habits on AL, SEMs were estimated separately for traumatic and psychosocial stressors, with perceived influence over the last year and resilience as mediators. The traumatic stressors model shows satisfactory fit statistics (*χ*^2^(7) = 7.448, *p* = 0.384; TLI = 0.997, CFI = 1.00, RMSEA = 0.01, SRMR = 0.0168). For the psychosocial stressors model an additional direct path from years of education to AL was included to improve model fit (modification index = 4.345; model fit: *χ*^2^(6) = 10.83, *p* = 0.094; TLI = 0.963, CFI = 0.995, RMSEA = 0.036, SRMR = 0.0231).

**Figure 3:**
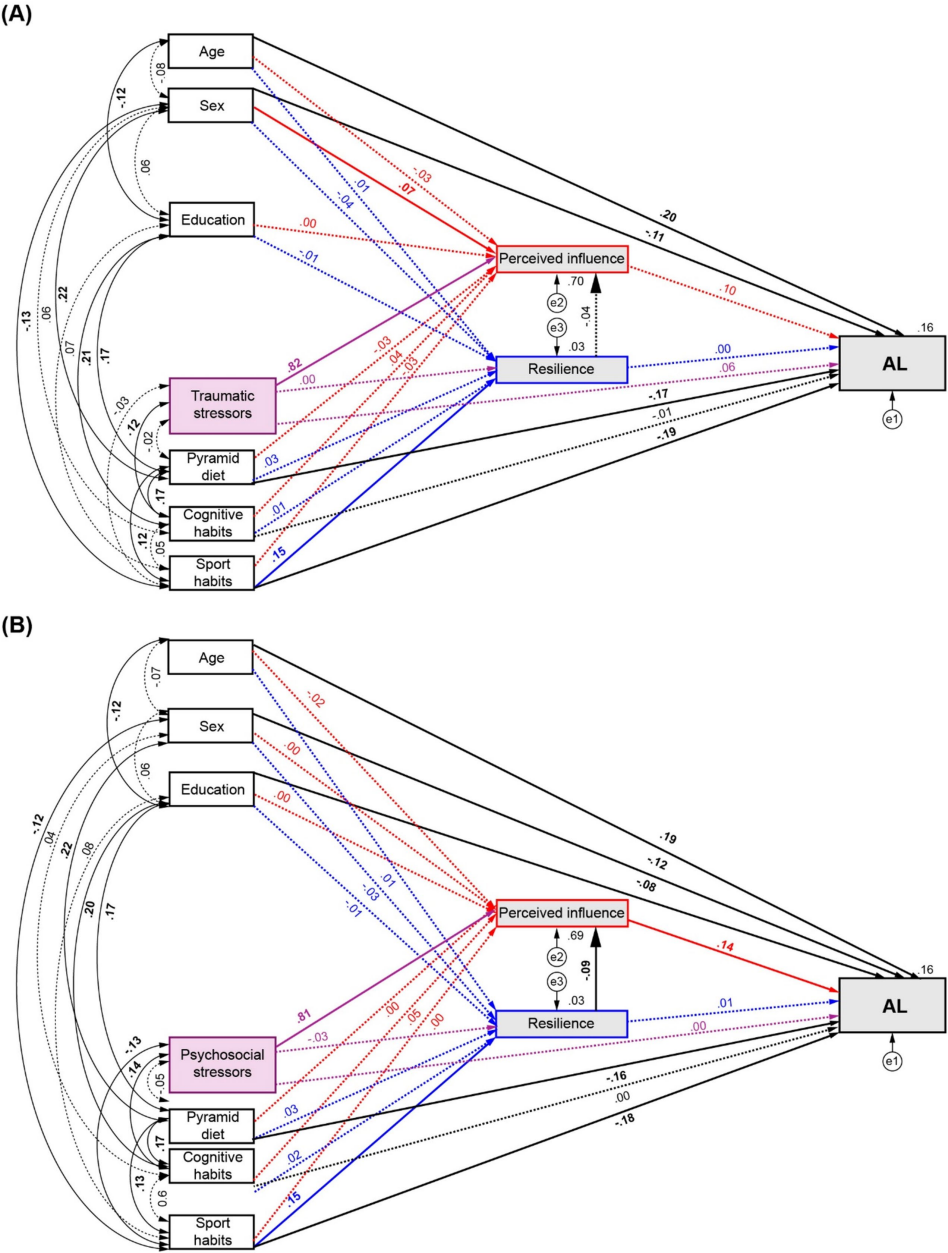
Path diagrams of the modulation of AL by number of stressors and healthy habits by mediation of perceived influence and resilience. **(A)** Effects of traumatic stressors and, **(B)** Effects of psychosocial stressors and healthy habits on AL. Red arrows: direct effects to and from perceived influence. Blue arrows: direct effects to and from resilience. Purple arrows: direct effects from number of stressors. Significant effects are shown by continuous lines. Standardised regression coefficients, covariances and R^2^ values for AL and mediator are reported.

Figure 3A shows the path diagram of the modulation of AL by traumatic stressors and healthy habits by mediation of perceived influence and resilience. Significant direct effects on AL were found from Pyramid scores (*β* = −0.171, *p* = 0.002, 95% CI = [−0.239,-0.093]) and sports habits (*β* = −0.189, *p* = 0.002, 95% CI = [−0.269,-0.111]). The latter also exerted a significant direct effect on resilience (*β* = 0.152, *p* = 0.003, 95% CI = [0.061, 0.234]). Significant direct effects were also found from resilience to perceived influence (*β* = −0.042, *p* = 0.036, 95% CI = [−0.085,-0.004]), but not from perceived influence on AL (*β* = −0.103, *p* = 0.203, 95% CI = [−0.067, 0.238]) or from resilience to AL (*β* = 0.001, *p* = 0.982, 95% CI = [−0.07, 0.076]). None of the selected mediation paths revealed significant indirect effects (Full statistical details described in Supplementary Table S8).

The assessment of psychosocial stressors through Model 1 (Figure 3B) showed equivalent direct effects of diet (*β* = −0.156, *p* = 0.001, 95% CI = [−0.231, −0.08]) and sports habits (*β* = −0.178, *p* = 0.003, 95% CI = [−0.254, −0.096]) on AL, and from sports habits to resilience (*β* = 0.148, *p* = 0.003, 95% CI = [0.049, 0.229]). Significant direct effects were also found from resilience to perceived influence (*β* = −0.088, *p* = 0.001, 95% CI = [−0.137, −0.039]), but not from perceived influence on AL (*β* = 0.141, *p* = 0.07, 95% CI = [−0.015, 0.299]) or from resilience to AL (*β* = 0.013, *p* = 0.739, 95% CI = [−0.065, 0.087]). A partial mediation on AL by sports habits was revealed by a significant indirect effect (*β* = −0.002, *p* = 0.027, 95% CI = [−0.006, 0]) through the third analysed pathway (*←Resilience ←perceived influence ← AL*). Full statistical details are summarised in Supplementary Table S9.

### 3.4 Sleep quality as pivotal mediator between life stressors and healthy habits on AL

Given that the analyses conducted through Model 1 did not reveal any direct or mediated associations between number of stressors and AL, we hypothesised that an additional factor could be underlying this effect. According to the reviewed evidence, sleep quality was chosen as an additional mediator between the effects of both perceived influence and resilience on AL to conduct a further assessment. In this second model, we hypothesised that the effect of life stressors and healthy habits mediated by perceived influence and resilience would modulate AL by affecting sleep quality. Satisfactory fit statistics were achieved for the sleep-modulated models of traumatic stressors (*χ*^2^(14) = 17.385, *p* = 0.236; TLI = 0.987, CFI = 0.997, RMSEA = 0.02, SRMR = 0.0193) and psychosocial stressors (*χ*^2^(14) = 18.999, *p* = 0.165; TLI = 0.981, CFI = 0.995, RMSEA = 0.024, SRMR = 0.0253).

**Figure 4:**
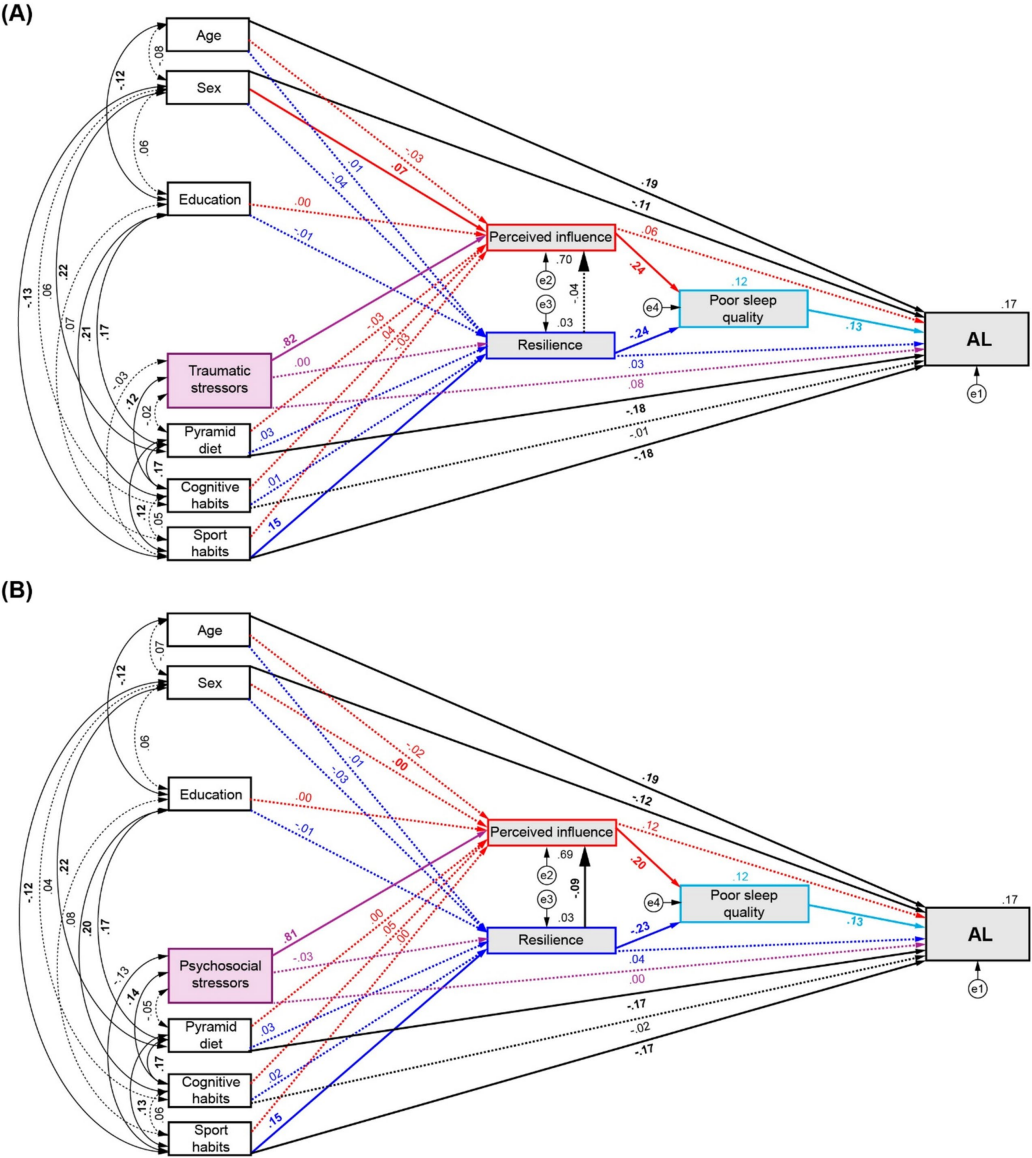
Path diagrams of the modulation of AL by number of stressors and healthy habits by mediation of perceived influence, resilience, and poor sleep quality. **(A)** Effects of traumatic stressors and, **(B)** Effects of psychosocial stressors and healthy habits on AL. Red arrows: direct effects to and from perceived influence. Blue arrows: direct effects to and from resilience. Purple arrows: direct effects from number of stressors. Significant effects are shown by continuous lines. Standardised regression coefficients, covariances and *R*^2^ values for AL and mediator are reported.

For traumatic stressors (Figure 4A), the SEM analysis confirmed significant associations between Pyramid scores and sports habits with AL (*β* = −0.176, *p* = 0.002, 95% CI = [−0.248, −0.099] and *β* = −0.181, *p* = 0.002, 95% CI = [−0.262, −0.103], respectively), and from sports habits to resilience (*β* = 0.152, *p* = 0.003, 95% CI = [0.061, 0.234]). Moreover, a strong effect of poor sleep quality on AL was found (*β* = 0.127, *p* = 0.002, 95% CI = [0.05, 0.202]), and equivalent but opposed direct effects on poor sleep quality emerged from perceived influence (*β* = 0.243, *p* = 0.001, 95% CI = [0.14,0.347]) and resilience (*β* = −0.243 *p* = 0.003, 95% CI = [−0.325, −0.152]). A full mediation effect of the number of traumatic stressors on AL was found (*β* = 0.025, *p* = 0.001, 95% CI = [0.01,0.05]) through the fourth path analysed (*←Perceived Influence ←Sleep ←AL*). A partial mediation effect of sports habits on AL was also revealed through the fifth (*←Resilience ←Sleep ←AL*) and sixth (*←Resilience ←Perceived Influence ←Sleep ←AL*) evaluated pathways (*β* = −0.005, *p* = 0.001, 95% CI = [−0.011,-0.002]; *β* = 0.000, *p* = 0.008, 95% CI = [−0.001, 0], respectively). For complete statistical details see Supplementary Table S10.

Similarly, when sleep was included in the path analysis for psychosocial stressors (Figure 4B), significant direct effects on AL were found from the Pyramid score and sports habits (*β* = −0.172, *p* = 0.002, 95% CI = [−0.242, −0.095]; *β* = −0.174, *p* = 0.003, 95% CI = [−0.253, −0.094], respectively), as also shown in the assessment of the first model. The direct effect of sports habits on resilience was also confirmed (*β* = 0.148, *p* = 0.003, 95% CI = [0.049, 0.229]) and the partial mediation effect of sport on AL by perceived influence and resilience shown in the analysis of Model 1 now appear relayed via sleep quality through the fifth (*β* = −0.004, *p* = 0.001, 95% CI = [−0.01, −0.002]) and sixth evaluated pathways (*β* = 0.000, *p* < 0.001, 95% CI = [−0.001, 0]). Additionally, full mediation effects were found on AL through the perceived influence to sleep pathway from the number of psychosocial stressors (*β* = 0.021, *p* = 0.001, 95% CI = [0.008, 0.04]) and from cognitive habits (*β* = 0.001, *p* = 0.033, 95% CI = [0, 0.004]). Full statistical details are described in Supplementary Table S11.

## 4 Discussion

### 4.1 Our findings

As previously reported, higher AL levels were shown in males, older ages and those with fewer years of education (Buller-Peralta et al., 2024). Whereas higher engagement in sports was correlated to males, females correlated to higher Pyramid scores, and number and perceived influence of psychosocial stressors. Similar differences in healthy habits have been found previously in adolescents, midlife and older adult studies, with females more engaged in healthier diet and males in sports and physical activities (Li et al., 2017; Sood et al., 2019; Boraita et al., 2020). Additionally, higher psychosocial stress perception, emerged from family, job, finances or health issues, have been reported in females compared to males (Costa et al., 2021; Méndez-Chacón, 2022).

None of the healthy habits analysed or types of stressors showed correlations with age, whereas more education correlated with younger participants, engagement in cognitive habits, adherence to Mediterranean diet and, weaker but significantly to sport habits. These results agree with previous evidence showing that more educated people tend to adopt healthier lifestyle habits, particularly regarding diet and physical activity (Gidlow et al., 2006; Kari et al., 2020; Azizi Fard et al., 2021). Additionally, adherence to a Mediterranean diet was correlated with more frequent engagement in cognitive and sports activities, which could be explained by the significative relation between level of education and engagement in healthy lifestyles.

Also, in line with previous studies, lower levels of AL were correlated with higher Pyramid diet scores (Mattei et al., 2013; Beydoun et al., 2019; Obomsawin et al., 2022; Zhou et al., 2022), frequent engagement in sports (Gay et al., 2015; Upchurch et al., 2015; Petrovic et al., 2016; Zhang et al., 2022; Bu and Li, 2023) and better sleep quality (McEwen, 2006; Clark et al., 2014; Bei et al., 2017; Hux et al., 2017; Christensen et al., 2022).

Number of traumatic stressors were positively correlated with psychosocial events, as well as between their respective perceived influence. This may suggest that regardless of the type of stressor, the more events experienced in life, the greater distress will be perceived at present.

A higher number of both traumatic and psychosocial stressors, and their perceived influence, correlated with increased engagement in cognitive activities and poorer sleep quality. However, lower number and influence of psychosocial stressors correlated with increase sports practice, consistent with sex differences favouring males in these factors.

Sleep quality showed no correlation with sex, age or years of education. However, greater engagement in sports was the only healthy habit correlated with better sleep quality and resilience abilities. Similar finding relating physical activity and sleep quality improvement have been extensively reported both in healthy children, adolescents and older adults (Baron et al., 2013; Rosa et al., 2021; Chen et al., 2024), and people with impaired sleep such as insomnia or major depressive disorder (Xie et al., 2021; Khazaie et al., 2023; Chen et al., 2024).

In our first analysis for traumatic stressor and AL mediated by resilience and perceived influence (Model 1), no effects of trauma were found, although significant direct effects on AL were shown from Pyramid diet scores and sport habits. The latter also exerted a significant direct effect on resilience. Similar results were found in the path analysis for psychosocial stressors through Model 1. Both results confirm previous findings associating sports and healthy diet with low AL levels and suggest that these habits could be preventing increased inflammatory, cardiovascular and metabolic factors, regardless of the influence from traumatic or psychosocial stressors. Interestingly, resilience showed a significant effect on the perceived influence of both psychosocial stressors and traumatic stressors, suggesting a potential impairment of resilience abilities after trauma as reported in PTSD (Jovanovic et al., 2012; Knox et al., 2012; Garfinkel et al., 2014; Lissek and van Meurs, 2015). Moreover, engagement in sport habits also showed a positive association with resilience and exerted a significant indirect effect on AL through the relation between resilience and lower perceived influence of psychosocial stressors. Recent evidence reports similar enhancing effects on resilience by physical activity practice (Antonini Philippe et al., 2021; Arida and Teixeira-Machado, 2021; Lancaster and Callaghan, 2022; Neumann et al., 2022).

Our second analysis of the effects of traumatic stressors on AL with sleep quality as additional mediator (Model 2) confirmed the significant associations between Pyramid score and sport habits on AL, and revealed a strong effect of sleep on AL, with poorer sleep quality associated with higher AL levels. Interestingly, opposed direct effects on poor sleep quality emerged from perceived influence and resilience, suggesting that the modulation of traumatic stress on AL might rely on a proportional balance between resilience ability and perceived influence over sleep quality. As previously reported, sleep is essential to adaptive processing of emotional experiences (Walker and van der Helm, 2009; Vandekerckhove and Cluydts, 2010; Tempesta et al., 2018; Rho et al., 2023). It should therefore not be surprising to unveil an effect of traumatic stressors when sleep is included as additional mediator since a bidirectional relationship between impaired sleep and PTSD has been extensively studied (Maher et al., 2006; Mellman et al., 2007; Lancel et al., 2021), as well as the role it plays in promoting resilience (Arora et al., 2022) and extinction of learned fear after traumatic events (Marshall et al., 2014; Genzel et al., 2015). Moreover, experimental evidence obtained through sleep deprivation protocols have shown that poor sleep quality can be considered as a stressor by itself (McEwen, 2006) that promotes high AL levels by increasing blood pressure, cortisol release, glucose resistance and inflammatory markers, among other effects (Spiegel et al., 1999; Knutson, 2007; Palagini et al., 2013; Dzierzewski et al., 2020; Li and Shang, 2021).

The full mediation effect found between the number of traumatic stressors on AL through associations between perceived influence and sleep quality also agrees with studies on PTSD, where resilience ability is impaired to extinguish the perceived effects of traumatic stressors, In the absence of additional healthy habits modulators the amount of experienced traumatic events tips the balance towards increased levels of AL by outweighing the protective effect of resilience. Additionally, when sleep was added to the analysis of traumatic stressors, a partial mediation of sport habits on AL was revealed through a positive association to resilience, through both a direct effect of resilience on sleep and via perceived influence on sleep. Thus, the engagement in sports habits not only decreases AL by itself, but it can also potentiate the protective sleep-mediated effects of resilience on AL levels directly, and by tipping the balance against the effect of perceived traumatic stress on AL through poor sleep quality.

When Model 2 was assessed for psychosocial stressor, the associations of diet and sport habits on AL, and the direct effect of sports on resilience shown by the first analysis were confirmed. A full mediation of the number of psychosocial stressors on AL was also unveiled through the association of perceived influence on sleep, in an equivalent magnitude to that seen for the case of the numbers of traumatic stressors, and in agreement with evidence reporting that sleep is affected by repeated stress regardless the kind of events experienced (Jean Kant et al., 1995; Van Reeth et al., 2000; Kim and Dimsdale, 2007; Mellman et al., 2007; Pawlyk et al., 2008; Ackermann et al., 2019; Lancel et al., 2021).

Interestingly, a full mediation effect of cognitive habits on AL was found through positive association of perceived influence and sleep, suggesting that higher engagement in cognitive activities increases perceived influence of psychosocial stressors and worsen sleep quality. This result contradicts existing evidence associating lower AL with practice of cognitive lifestyle habits (García-Moreno et al., 2021; Wang et al., 2021), and no studies have yet described a similar finding. However, such relation could be explained in part by the significant positive correlation found between engagement in cognitive activities and perceived influence of psychosocial stressors (*r* = 0.159, *p* < 0.001). If we consider frequent involvement in cognitively stimulating activities as an indicator of higher intelligence, some studies have shown a positive association between that and self-awareness, worry, rumination and anxiety levels (Coplan et al., 2012; Penney et al., 2015; Sutton, 2016; du Pont et al., 2020), suggesting that more intellectual individuals are more prone to develop rumination of negative experiences as an adaptive process to enhance problem solving (Andrews and Thomson, 2009), causing them frequent negative repetitive thoughts that may increase their experience of emotional distress (Constantin et al., 2021).

Finally, the partial mediation effect of sport habits on AL by perceived influence and resilience shown in the first analysis now appear relayed via sleep quality, in a similar way as shown in the assessment of traumatic stressors, through both a direct effect of resilience on sleep and via perceived influence on sleep. These results support the idea regarding the enhancement of resilience by physical activity and its protective on AL mediated by sleep, regardless of the type of stressor experienced. Altogether, the analyses of traumatic and psychosocial events through the second model revealed the pivotal role of sleep in the modulation of AL by healthy habits and life stressors.

### 4.2 Conclusion and limitations

**Figure 5:**
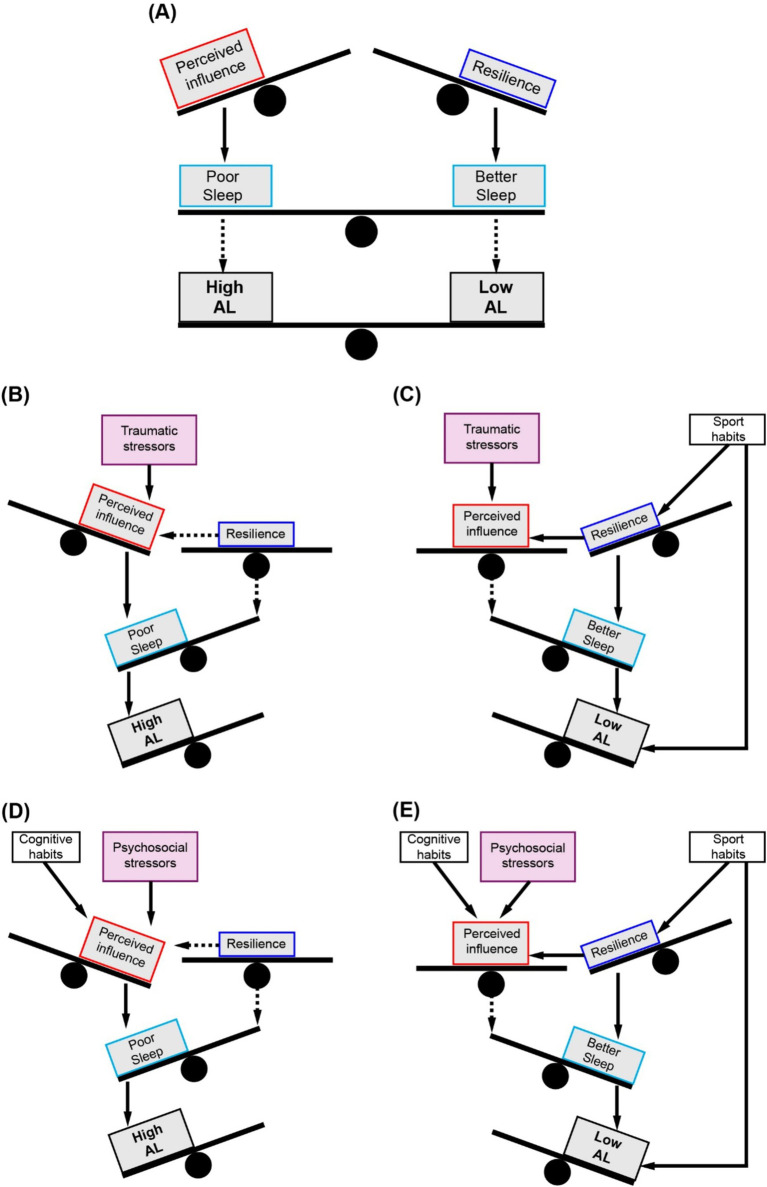
Balance model of AL modulation through sleep mediation. **(A)** Proportional balance in the effects of perceived influence of stressors and resilience on sleep quality, modulating AL. **(B)** Traumatic stressors tip the balance towards high AL by worsening sleep. **(C)** Sport habits compensate the balance towards low AL directly and by improving sleep through enhanced resilience. **(D)** Psychosocial stressors and cognitive habits tip the balance towards high AL by worsening sleep. **(E)** Sport habits compensate the balance towards low AL directly and by improving sleep through enhanced resilience.

As mentioned at the beginning of this work, high AL levels are caused by three possible situations: *(i)* a repeated stress exposure, reflected in our analyses by the number of life stressors; *(ii)* the inability to successfully adapt to stress demands through effective copying strategies, assessed through the resilience questionnaire; and *(iii)* the inability to terminate the physiological response to stress after the end of the event, also assessed through resilience levels and its relation to the amount of influence of the stressors perceived during the past year. Only by including sleep as mediator of the associations between life stressors and healthy habits on AL, the modulation by the three situations emerged: *(i)* the first through the effects of the number of events, whether traumatic or psychosocial; *(ii)* the second by the protective effect of resilience on AL by improving sleep quality; and *(iii)* the third, by increasing the weight of the association between perceived influence on poor sleep and surpassing the effect of resilience, in the case of traumatic stressors. This suggested a hypothetical proportional balance in the effects of perceived influence of stressors and resilience on sleep quality, such that the former acts to worsen it while the latter improves it (Figure 5A). This balance mediates the modulation of AL levels by weighing the influence of stressors (Figures 5B,C) and healthy habits, tipping towards decreased AL when frequent engagement in sports practice is added to the equation (Figures 5D,E).

However, a word of caution regarding these conclusions should be mentioned, as our results rely on a self-reported sleep questionnaire and not through objective sleep measurements. Previous studies have shown that self-reported perception of health and well-being could be negatively biased in stressed and highly deprived populations (Loughnan et al., 2011; Bradford et al., 2023), pointing to the need to use more objective sleep measurements to further validate our analysis. As gold-standard polysomnography is unfeasible to be performed in large samples of participants, actigraphy measurements obtained through by wearable devices emerge as an option to consider in future assessments, not only because of their proved reliability but also due to the possibility to evaluate further sleep structures such as NREM and REM sleep amounts (Etindele Sosso et al., 2021; Yuan et al., 2024).

## Data Availability

Publicly available datasets were analyzed in this study. This data can be found here: The PREVENT dataset is available to access through a data request on the study website (www.preventdementia.co.uk); on the Alzheimer’s Disease Data Initiative (ADDI) platform baseline dataset DOI: https://doi.org/10.34688/PREVENTMAIN_BASELINE_700V1; Dementia Platforms UK (DPUK); and the Global Alzheimer’s Association Network (GAAIN).
