# Supplementary material for: The pivotal role of sleep in mediating the effects of life stressors and healthy habits on allostatic load in mid-life adults
Source: Front Hum Neurosci. 2024 Dec 20;18:1509223. doi: 10.3389/fnhum.2024.1509223 (PMC11695329; doi:10.3389/fnhum.2024.1509223)
Supplement: Supplementary file 1 [file Data_Sheet_1.pdf]

Supplementary Material

The pivotal role of sleep in mediating the effects of life stressors and healthy habits on allostatic load in mid-life adults.

- **Supplementary Figure S1.** Algorithm flowchart for comprehensive AL scoring (ALCS) construction
- **Supplementary Table S1.** Cut-off thresholds for Allostatic Load (AL) scorings
- **Supplementary Text S1.** Items selected as traumatic or psychosocial stressors from the Life Stressor Checklist-Revised (LSC-R)
- **Supplementary Table S2.** Pyramid diet scoring criteria.
- **Supplementary Text S2.** Items selected for cognitive and healthy habits evaluation from the Lifetime of Experiences Questionnaire (LEQ)
- **Supplementary Table S3.** Statistical comparisons between males and females
- **Supplementary Table S4.** Assessment of normality and multivariate normality of variables and models
- **Supplementary Table S5.** Multicollinearity and linearity assumption tests
- **Supplementary Table S6.** Logistic regression assessment for associations between full data set and Pyramid Score missing values
- **Supplementary Table S7.** Bivariate correlations
- **Supplementary Table S8.** Direct and indirect effects of AL modulation by traumatic life stressors and healthy habits mediated by perceived influence and resilience.
- **Supplementary Table S9.** Direct and indirect effects of AL modulation by psychosocial life stressors and healthy habits mediated by perceived influence and resilience.
- **Supplementary Table S10.** Direct and indirect effects of AL modulation by traumatic life stressors and healthy habits mediated by perceived influence, resilience, and poor sleep quality.
- **Supplementary Table S11.** Direct and indirect effects of AL modulation by psychosocial life stressors and healthy habits mediated by perceived influence, resilience, and poor sleep quality.

Corresponding Author: Ingrid Buller-Peralta (ingrid.buller@ed.ac.uk). Edinburgh Dementia Prevention - Centre for Clinical Brain Sciences, The University of Edinburgh. Outpatients Department Level 2 Western General Hospital, Crewe Rd S, Edinburgh, EH4 2XU, UK.

Supplementary Figure S1. Algorithm flowchart for comprehensive AL scoring (ALCS) construction.

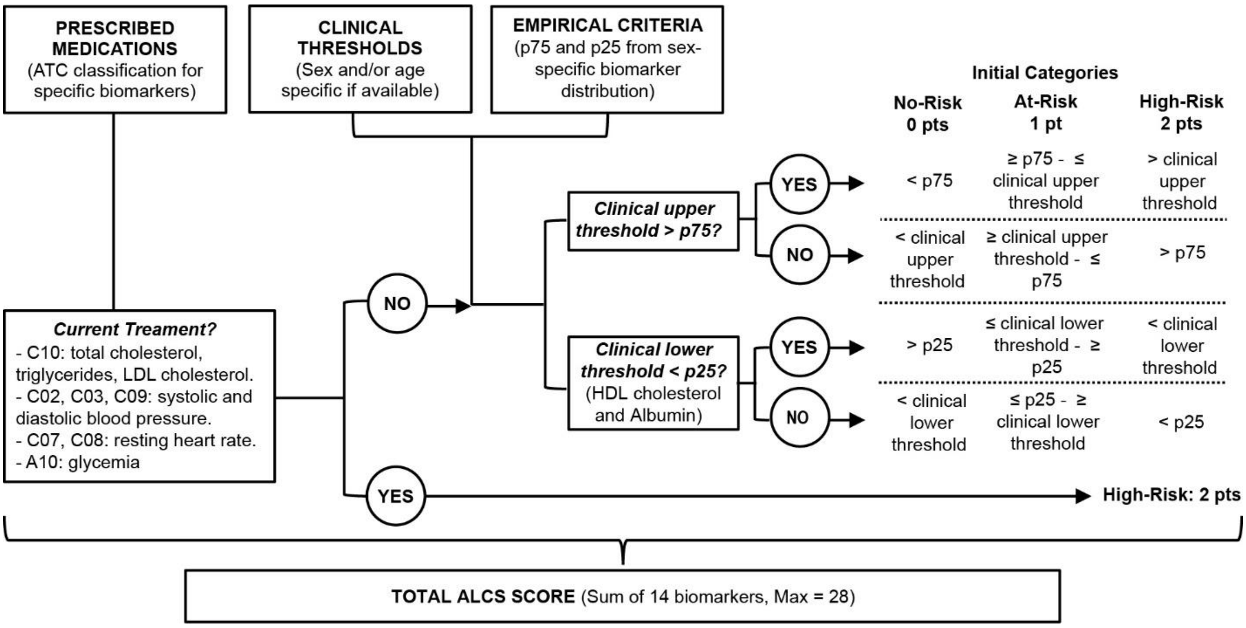

Supplementary Table S1. Cut-off thresholds for Allostatic Load (AL) scorings							
System	Biomarker	Sex	Clinical thresholds	p75 (p25*)	Risk categories		
					No-Risk	*At-Risk*	*High-Risk*
Immune	**Creatinine [μmol/L]**	Male	59 - 104	88.00	≤ 87.99	≥88 - ≤104	≥ 104.01
		Female	45 - 84	69.00	≤ 68.99	≥69 - ≤84	≥ 84.01
	**Albumin* [gr/L]**	Male	35 - 50	40.00	≥ 40.01	≤35 - ≥40	≤ 34.99
		Female	same as Male	38.00	≥ 38.01	≤35 - ≥38	≤ 34.99
	**C-reactive protein (CRP) [mg/L]**	Male	<5	3.65	≤ 3.64	≥3.65 - ≤4.99	≥ 5
		Female	same as Male	4.00	≤ 3.99	≥4 - ≤4.99	≥ 5
	**Fibrinogen [gr/L]**	Male	1.7 – 4.98	3.10	≤ 3.09	≥3.1 - ≤4.98	≥ 4.99
		Female	same as Male	3.38	≤ 3.37	≥3.38 - ≤4.98	≥ 4.99
Metabolic	**Total Cholesterol [mmol/L]**	Male	0 - 5.0	5.94	≤ 5	≥5.01 - ≤5.94	≥ 5.95
		Female	same as Male	6.19	≤ 5	≥5.01 - ≤6.19	≥ 6.2
	**HDL Cholesterol* [mmol/L]**	Male	1.0 – 3.0	1.17	≥ 1.18	≥1 - ≤1.17	≥ 0.99
		Female	same as Male	1.48	≥ 1.49	≤1 - ≥1.48	≤ 0.99
	**LDL Cholesterol [mmol/L]**	Male	1.0 – 3.0	3.8	≤ 3	≤3.01 - ≥3.8	≤ 3.81
		Female	same as Male	3.83	≤ 3	≥3.01 - ≤3.83	≥ 3.84
	**Triglycerides [mmol/L]**	Male	0 – 1.7	1.6	≤ 1.59	≥1.6 - ≤1.7	≥ 1.71
		Female	same as Male	1.2	≤ 1.19	≥1.2 - ≤1.7	≥ 1.71
	**Glycemia [mmol/L]**	Male	5.5 - 6.9	5.4	≤ 5.39	≥5.4 - ≤6.9	≥ 6.91
		Female	same as Male	5.2	≤ 5.19	≥5.2 - ≤6.9	≥ 6.91
	**Body mass index (BMI) [kg/m2]**	Male	25 - 29.9	30.33	≤ 24.99	≥25 - ≤29.99	≥ 30
		Female	same as Male	30.22	≤ 24.99	≥25 - ≤29.99	≥ 30
Cardiovascular	**Systolic blood pressure (SBP) [mmHg]**	Male	120 - 149	140.5	≤ 140.49	≥140.5 - ≤149	≥ 149.01
		Female	same as Male	127.67	≤ 127.66	≥127.67 - ≤149	≥ 149.01
	**Diastolic blood pressure (DBP) [mmHg]**	Male	80 - 89	86.17	≤ 86.16	≥86.17 - ≤89	≥ 89.01
		Female	same as Male	79	≤ 78.99	≥79 - ≤89	≥ 89.01
	**Waist-to-hip ratio (WHR)**	Male	no-risk ≤0.95, risk: 0.96-0.99, high-risk ≥1	0.98	≤ 95.99	≥96 - ≤0.99	≥ 1
		Female	no-risk ≤0.80, risk: 0.81-0.84, high-risk ≥85	0.87	≤ 80.99	≥0.81 - ≤0.84	≥ 85
	**Resting heart rate (RHR) [BPM]**						
	Age ≤45	Male	average: <76, below average: 76-82, poor:>82	69.67	≤ 75.99	≥76 - ≤82	≥ 82.01
		Female	average: <79, below average: 79-84, poor:>84	69.83	≤ 78.99	≥79 - ≤84	≥ 84.01
	Age 46 - 55	Male	average: <77, below average: 77-83, poor:>83	67	≤ 76.99	≥77 - ≤83	≥ 83.01
		Female	average: <78, below average: 78-83, poor:>83	69.67	≤ 77.99	≥78 - ≤83	≥ 83.01
	Age 56 - 65	Male	average: <76, below average: 76-81, poor:>81	67	≤ 75.99	≥76 - ≤81	≥ 81.01

		Female	average: <78, below average: 78-83, poor:>83	69.67	≤ 77.99	≥78 - ≤83	≥ 83.01
* Reverse scorings (p25 or low clinical thresholds used for risk)							

Supplementary Text S1. Items selected as traumatic or psychosocial stressors from the Life Stressor Checklist - Revised (LSC-R)
(* = Traumatic; ǂ = Psychosocial; [N/I] = Not included)

* 1. Have you ever been in a serious disaster (for example, an earthquake, hurricane, large fire, explosion)?
* 2. Have you ever seen a serious accident (for example, a bad car wreck or an on-the-job accident)?
* 3. Have you ever had a very serious accident or accident-related injury (for example, a bad car wreck or an on-the-job accident)?

ǂ 4. Was a close family member ever sent to jail?
ǂ 5. Have you ever been sent to jail?
ǂ 6. Were you ever put in foster care or put up for adoption?
ǂ 7. Did your parents ever separate or divorce while you were living with them?
ǂ 8. Have you ever been separated or divorced?
ǂ 9. Have you ever had serious money problems (for example, not enough money for food or place to live?
ǂ 10. Have you ever had a very serious physical or mental illness (for example, cancer, heart attack, serious operation, felt like killing yourself, hospitalized because of nerve problems?
ǂ 11. Have you ever been emotionally abused or neglected (for example, being frequently shamed, embarrassed, ignored, or repeatedly told that you were “no good”)?

* 12. Have you ever been physically neglected (for example, not fed, not properly clothed, or left to take care of yourself when you were too young or ill)?
* 13. WOMEN ONLY: Have you ever had an abortion or miscarriage (lost your baby)?

ǂ 14. Have you ever been separated from your child against your will (for example, the loss of custody or visitation or kidnapping)?
ǂ 15. Has a baby or child of yours ever had a severe physical or mental illness (for example, mentally retarded, birth defects, can’t hear, see, walk)?
ǂ 16. Have you ever been responsible for taking care of someone close to you (not your child) who had a severe physical or mental handicap (for example, cancer, stroke, AIDS, nerve problems, can’t see, walk)?

* 17. Has someone close to you died suddenly or unexpectedly (for example, sudden heart attack, murder, suicide)?

ǂ 18. Has someone close to you died (do NOT include those who died suddenly or unexpectedly)?

* 19. When you were young (before age 16). Did you ever see violence between family members (for example, hitting, kicking, slapping, punching)?

* 20. Have you ever seen a robbery, mugging, or attack taking place?
* 21. Have you ever been robbed, mugged, or physically attacked (not sexually) by someone you did not know?
* 22. Before age 16, were you ever abused or physically attacked (not sexually) by someone you knew (for example, a parent, boyfriend, or husband, hit, slapped, choked, burned, or beat you up?
* 23. After age 16, were you ever abused or physically attacked (not sexually) by someone you knew (for example, a parent, boyfriend, or husband, hit, slapped, choked, burned, or beat you up?

ǂ 24. Have you ever been bothered or harassed by sexual remarks, jokes, or demands for sexual favours by someone at work or school (for example, a coworker, a boss, a customer, another student, a teacher)?

* 25. Before age 16, were you ever touched or made to touch someone else in a sexual way because he/she forced you in some way or threatened to harm you if you didn't?
* 26. After age 16, were you ever touched or made to touch someone else in a sexual way because he/she forced you in some way or threatened to harm you if you didn't?
* 27. Before age 16, did you ever have sex (oral, anal, genital) when you didn't want to because someone forced you in some way or threatened to hurt you if you didn't?
* 28. After age 16, did you ever have sex (oral, anal, genital) when you didn't want to because someone forced you in some way or threatened to hurt you if you didn't?

[N/I] 29. Are there any events we did not include that you would like to mention?

[N/I] 30. Have any of the events mentioned above ever happened to someone close to you so that even though you didn't see it yourself, you were seriously upset by it?

Supplementary Table S2. Pyramid diet scoring criteria[a]

Food component	Pyramid rule	Pyramid score
Olive oil (consumption)	Rule 15	1 point: Consumption
		0 points: Non-consumption
Vegetables	Rule 1	1 point: ≥6 portions /day
		0 points: 0 portions /day
Fruit	Rule 3	1 point: 3 - 6portions /day
		0 points: 0 portions /day
		0.5 points: overconsumption [b]
Red meat	Rule 8	1 point: <2 portion /day
		0 points: ≥2 portions /week
Dairy	Rule 6	1 point: 1.2 - 2.5 portions /d
		0 point: 0 portions
		0.5: overconsumption [b]
Alcohol	Rule 14	1 point: 1.5 - 2.5 portions /day (male)
		1 point: 0.5 - 1.5 portions /day (female)
		0 points: 0 portions /day
		0.5: overconsumption [b]
Pulses	Rule 2	1 point: ≥2 portions /week
		0 points: 0 portions /week
Fish / seafood	Rule 7	1 point: ≥2 portions /week
		0 points: 0 portions /week
Nuts	Rule 4	1 point: 1-2 portions /day
		0 points: 0 portions /day
		0.5 points: overconsumption [b]
White meat	Rule 10	1 point: 1.5 - 2.5 portions /week
		0 point: 0 portions /week
		0.5: overconsumption [b]
Cereals	Rule 5	1 point: 3-6 portions /day
		0 points: 0 portions /day
		0.5 points: overconsumption [b]
Processed meat	Rule 9	1 point: <2 portion /week
		0 points: ≥2 portions /week
Eggs	Rule 11	1 point: 2-3 portions /week
		0 points: portions /week
		0.5: overconsumption [b]
Potato	Rule 12	1 point: ≤3 portions /week
		0 points: >3 portions /week
Sweets	Rule 13	1 point: ≤2 portions /week
		0 points: >2 portions /week

[a] Adapted from *Gregory et al, 2023*.
[b] Two-fold higher than mid-point of recommended intake.

Supplementary Text S2. Items selected for cognitive and healthy habits evaluation from the Lifetime of Experiences Questionnaire (LEQ).

Scoring: frequency of engagement during their middle age years, after the age of 30 until the end of working life or present (0= never, 1= less than monthly, 2= monthly, 3= fortnightly, 4= weekly, 5= daily).

Cognitive habits (4 items selected, max score = 20)
1. How often were you practicing or playing a musical instrument?
2. How often would you practice or develop an artistic pastime (e.g. drawing, painting, writing, acting)?
3. How often did you read (material of any sort) for more than five minutes?
4. How often would you practice speaking a second language?

Sport habits (3 items selected, max score = 15)
1. How often would you take part in sports or activities that are mildly energetic (e.g. walking, woodwork, weeding, hoeing, bicycle repair, playing pool, general housework)?
2. How often would you take part in sports or activities that are moderately energetic (e.g. scrubbing, polishing car, dancing, golf, cycling, decorating, lawn mowing, leisurely swimming)?
3. How often would you take part in sports or activities that are vigorous (e.g. running, hard swimming, tennis, squash, digging, cycle racing)?

Supplementary Table S3. Statistical comparisons between males and females				
		**Mean ± SD**	**Normality**[a]	**Independent samples test**[b]
**Age**	Males	51.71 ± 5.56	W=0.931, p<0.001	U=41731.5, p=0.07
	Females	50.97 ± 5.41	W=0.951, p<0.001	
**Years Education**	Males	16.35 ± 3.2	W=0.959, p<0.001	U=47973, p=0.287
	Females	16.75 ± 3.68	W=0.957, p<0.001	
**Allostatic load score**	Males	6.88 ± 3.99	W=0.939, p<0.001	U=38615.5, p=0.001 **
	Females	5.79 ± 3.83	W=0.945, p<0.001	
**Pyramid diet score**	Males	7.71 ± 1.43	W=0.992, p=0.260	U=29140, p<0.001 ***
	Females	8.4 ± 1.51	W=0.993, p=0.128	
**Cognitive habits**	Males	7.17 ± 3.12	W=0.869, p<0.001	U=42348.5, p=0.122
	Females	7.5 ± 2.92	W=0.917, p<0.001	
**Sport habits**	Males	11.29 ± 2.76	W=0.913, p<0.001	U=38698.5, p=0.001 **
	Females	10.54 ± 2.88	W=0.937, p<0.001	
**No traumatic stressors**	Males	2.54 ± 1.94	W=0.920, p<0.001	U=44241, p=0.505
	Females	2.46 ± 1.99	W=0.891, p<0.001	
**No psychosocial stressors**	Males	2.53 ± 1.73	W=0.891, p<0.001	U=40360.5, p=0.012 *
	Females	2.92 ± 1.87	W=0.911, p<0.001	
**Perceived influence traumatic stressors**	Males	4.12 ± 4.65	W=0.725, p<0.001	U=43083, p=0.231
	Females	4.61 ± 4.74	W=0.820, p<0.001	
**Perceived influence psychosocial stressors**	Males	4.34 ± 4.16	W=0.835, p<0.001	U=39931, p=0.008 **
	Females	5.18 ± 4.76	W=0.824, p<0.001	
**Resilience**	Males	73.61 ± 12.43	W=0.968, p<0.001	U=43353.5, p=0.287
	Females	72.33 ± 13.31	W=0.977, p<0.001	
**Poor sleep quality**	Males	5.84 ± 3.02	W=0.906, p<0.001	U=42556.5, p=0.149
	Females	6.16 ± 3.1	W=0.940, p<0.001	

[a] Shapiro-Wilk test

[b] 2-sided Mann-Whitney test (α=0.05). Significance: *p < 0.05; **p < 0.01; ***p < 0.001.

Supplementary Table S4. Assessment of normality and multivariate normality of variables and models

**Variable**	**Skewness**	**Kurtosis**
Age	-0.424	-0.886
Sex	-0.457	-1.792
Education	0.238	3.208
Allostatic Load	*0.775*	*0.217*
No Traumatic events	1.098	2.07
No Psychosocial events	0.984	1.013
Influence traumatic events	*2.325*	***8.89*** [a]
Influence psychosocial events	*1.801*	*3.965*
Pyramid diet	0.012	0.841
Cognitive habits	1.089	1.745
Sport habits	-0.772	0.202
Resilience	*-0.613*	*0.611*
Poor sleep quality	*0.979*	*0.904*

**Mardia's coefficient for multivariate kurtosis**	**Kurtosis**	**Critical ratio**
Model 1 Traumatic stressors	29.063	23.356
Model 1 Psychosocial stressors	16.213	13.03
Model 2 Traumatic stressors	31.122	22.911
Model 2 Psychosocial stressors	17.87	13.155

Endogenous variables in *italic* font

[a] Kurtosis value showing substantial deviation from normality (>8 for samples larger than n=300 (West et al., 1995; Kline, 2011).

Supplementary Table S5. Multicollinearity and linearity assumption tests

	Traumatic life stressors				Psychosocial life stressors			
	Multicollinearity		***Deviation for Linearity***		***Multicollinearity***		***Deviation for Linearity***	
	VIF	Tolerance	F ratio (df deviation,df within groups)	p	VIF	Tolerance	F ratio (df deviation,df within groups)	p
← **Perceived Influence**								
Number of Events	1.019	0.981	F(10,608)= 0.61	0.807	1.058	0.945	F(7,611)= 1.6	0.131
Pyramid Diet Score	1.121	0.892	F(617,1)= 2.78	0.451	1.124	0.89	F(617,1)= 10.4	0.243
Cognitive Habits	1.085	0.922	F(18,600)= 0.74	0.776	1.094	0.914	F(18,600)= 1.29	0.186
Sports Habits	1.054	0.949	F(14,604)= 0.64	0.831	1.071	0.934	F(14,604)= 1.25	0.235
Age	1.026	0.975	F(19,599)= 1.19	0.257	1.028	0.973	F(19,599)= 0.6	0.911
Sex	1.085	0.921	-	-	1.094	0.914	-	-
Education	1.087	0.92	F(20,598)= 0.57	0.936	1.083	0.923	F(20,598)= 1.07	0.378
← **Resilience**								
Number of Events	3.244	0.308	F(10,608)= 0.67	0.753	3.136	0.319	F(7,611)= 0.53	0.814
Pyramid Diet Score	1.125	0.889	F(617,1)= 0.43	0.873	1.124	0.89	§	§
Cognitive Habits	1.089	0.918	F(18,600)= 0.76	0.748	1.1	0.909	§	§
Sports Habits	1.057	0.946	F(14,604)= 1.45	0.126	1.071	0.933	§	§
Age	1.028	0.973	F(19,599)= 0.6	0.911	1.03	0.971	§	§
Sex	1.1	0.909	-	-	1.094	0.914	§	§
Education	1.087	0.92	F(20,598)= 1.02	0.437	1.083	0.923	§	§
Percieved influence	3.267	0.306	F(25,593)= 0.85	0.683	3.127	0.32	F(24,594)= 0.88	0.634
← **AL**								
Number of Events	3.286	0.304	F(10,608)= 1.21	0.279	3.173	0.315	F(7,611)= 0.81	0.58
Pyramid Diet Score	1.128	0.887	F(617,1)= 1.84	0.539	1.127	0.887	§	§
Cognitive Habits	1.09	0.918	F(18,600)= 1.62	0.051	1.101	0.909	§	§
Sports Habits	1.085	0.922	F(14,604)= 0.95	0.503	1.097	0.911	§	§
Age	1.029	0.972	F(19,599)= 0.66	0.862	1.03	0.971	§	§
Sex	1.101	0.909	-	-	1.095	0.913	§	§
Education	1.087	0.92	F(20,598)= 1.36	0.137	1.083	0.923	§	§
Percieved influence	3.444	0.29	F(25,593)= 1.46	0.071	3.242	0.308	F(24,594)= 0.75	0.797
Resilience	1.095	0.913	F(63,555)= 0.73	0.942	1.109	0.901	§	§
Sleep quality	1.16	0.862	F(16,602)= 0.45	0.969	1.123	0.89	§	§
← **Poor sleep quality**								
Percieved influence	1.002	0.998	F(25,593)= 1.49	0.059	1.018	0.983	F(24,594)= 1.08	0.364
Resilience	1.002	0.998	F(63,555)= 1.06	0.368	1.018	0.983	§	§

VIF: Variance inflation factor, §: Same results as those obtained in the Traumatic life stressors model assessment, given relations between same variables are assessed.

Supplementary Table S6. Logistic regression assessment for associations between full data set and Pyramid Score missing values

Reference category = non-missing pyramid score values[a]

**Model Fitting**	χ^2 (12)= 6.518, p=0.888				
**Pseudo R^2 [b]**	0.018				
**Parameter estimates**	**B**	**S.E**	**Exp(B)**	**p**	**95% CI for Exp(B)**
Age	0.016	0.03	1.016	0.578	0.961, 1.074
Sex	0.388	0.33	1.474	0.236	0.776, 2.801
Education	0.006	0.04	1.006	0.887	0.925, 1.095
Allostatic Load	0.035	0.04	1.036	0.384	0.957, 1.121
No Traumatic events	0.028	0.14	1.029	0.843	0.778, 1.361
No Psychosocial events	0.094	0.17	1.098	0.575	0.792, 1.523
Influence traumatic events	-0.039	0.07	0.962	0.555	0.846, 1.094
Influence psychosocial events	0.004	0.06	1.004	0.944	0.893, 1.13
Cognitive habits	-0.011	0.05	0.989	0.832	0.894, 1.094
Sport habits	0.066	0.06	1.068	0.255	0.954, 1.197
Resilience	-0.002	0.01	0.998	0.894	0.976, 1.022
Poor sleep quality	0.067	0.05	1.069	0.175	0.971, 1.178

B= regression coefficient, S.E. Standard error, Exp(B): Exponential regression coefficient, CI: confidence intervals for Exp(B) significance.

[a] Non-missing category = 0 (n=570), Missing category = 1 (n=50).

[b] Mc Fadden pseudo-R^2 reported.

Supplementary Table S7. Bivariate correlations[a]

	1	2	3	4	5	6	7	8	9	10	11	12	13
1. Sex	---												
2. Age	-0.065	---											
3. Years of education	0.055	**-0.118****	---										
4. Allostatic load	**-0.135****	**0.216*****	**-0.146*****	---									
5. Number traumatic stressors	-0.021	0.028	-0.042	**0.152*****	---								
6. Number psychosocial stressors	**0.105****	0.046	0.016	**0.142*****	**0.444*****	---							
7. Pyramid Diet	**0.217*****	0.002	**0.165*****	**-0.220*****	0.013	-0.026	---						
8. Cognitive habits	0.053	0.024	**0.204*****	-0.035	**0.110****	**0.143*****	**0.172*****	---					
9.Sport habits	**-0.127****	-0.058	**0.081***	**-0.217*****	-0.035	**-0.146*****	**0.125****	0.053	---				
10. Perceived influence traumatic stressors	0.051	-0.006	-0.024	**0.155*****	**0.828*****	**0.478*****	-0.007	**0.125****	-0.071	---			
11. Perceived influence psychosocial stressors	**0.090***	0.013	0.027	**0.158*****	**0.397*****	**0.823*****	-0.022	**0.159*****	**-0.132****	**0.545*****	---		
12. Resilience	-0.048	0.004	0.011	-0.036	0.002	-0.055	0.046	0.021	**0.160*****	-0.048	**-0.132****	---	
13. Poor sleep quality	0.05	0.02	-0.004	**0.161*****	**0.152*****	**0.182*****	0.019	0.021	**-0.120****	**0.255*****	**0.230*****	**-0.255*****	---

[a] Pearson correlation coefficients reported

2-tailed significance: *p < 0.05; **p < 0.01; ***p < 0.001.

Supplementary Table S8. Direct and indirect effects of AL modulation by traumatic life stressors and healthy habits mediated by perceived influence and resilience

		Direct Effects			Indirect Effects			
Modulator		Standardized (p)[a]	S.E[b]	95% CI[c]	***Mediation path***	Standardized (p)[a]	S.E[b]	95% CI[c]
Number of Events	← Perceived Influence	**0.824 (0.003)**	0.02	0.794, 0.852	← Perceived Influence ← AL	0.085 (0.201)	0.06	-0.053, 0.199
	← Resilience	0.004 (0.965)	0.04	-0.076, 0.081	← Resilience ← AL	0.000 (0.992)	0.00	-0.003, 0.003
	← AL	0.056 (0.44)	0.08	-0.083, 0.216	← Resilience ← Perceived Influence ← AL	0.000 (0.667)	0.00	-0.001, 0
Pyramid Diet Score	← Perceived Influence	-0.034 (0.121)	0.02	-0.082, 0.01	← Perceived Influence ← AL	-0.004 (0.114)	0.00	-0.018, 0.001
	← Resilience	0.034 (0.514)	0.05	-0.066, 0.12	← Resilience ← AL	0.000 (0.898)	0.00	-0.004, 0.005
	← AL	**-0.171 (0.002)**	0.04	-0.239, -0.093	← Resilience ← Perceived Influence ← AL	0.000 (0.24)	0.00	-0.001, 0
Cognitive Habits	← Perceived Influence	0.039 (0.129)	0.03	-0.01, 0.086	← Perceived Influence ← AL	0.004 (0.149)	0.00	-0.001, 0.017
	← Resilience	0.01 (0.806)	0.04	-0.067, 0.088	← Resilience ← AL	0.000 (0.983)	0.00	-0.003, 0.003
	← AL	-0.014 (0.786)	0.04	-0.085, 0.066	← Resilience ← Perceived Influence ← AL	0.000 (0.436)	0.00	-0.001, 0
Sports Habits	← Perceived Influence	-0.026 (0.31)	0.03	-0.076, 0.026	← Perceived Influence ← AL	-0.003 (0.201)	0.00	-0.016, 0.002
	← Resilience	**0.152 (0.003)**	0.04	0.061, 0.234	← Resilience ← AL	0.000 (0.982)	0.01	-0.012, 0.013
	← AL	**-0.189 (0.002)**	0.04	-0.269, -0.111	← Resilience ← Perceived Influence ← AL	-0.001 (0.098)	0.00	-0.003, 0
Age	← Perceived Influence	-0.026 (0.228)	0.02	-0.065, 0.02				
	← Resilience	0.01 (0.794)	0.04	-0.073, 0.084				
	← AL	**0.198 (0.002)**	0.04	0.122, 0.265				
Sex	← Perceived Influence	**0.065 (0.009)**	0.03	0.016, 0.113				
	← Resilience	-0.035 (0.357)	0.04	-0.12, 0.045				
	← AL	**-0.113 (0.003)**	0.04	-0.19, -0.035				
Education	← Perceived Influence	0.004 (0.927)	0.03	-0.049, 0.05				
	← Resilience	-0.006 (0.89)	0.04	-0.097, 0.077				
	← AL	Restricted	-	-				
	Perceived Influence ← Resilience	**-0.042 (0.036)**	0.02	-0.085, -0.004				
	AL← Perceived Influence	0.103 (0.203)	0.08	-0.067, 0.238				
	AL ← Resilience	0.001 (0.982)	0.04	-0.07, 0.076				

S.E: Standard Error, CI: confidence interval
[a] 2-Tailed Significance (Bias-Corrected)
[b] 2-Bias-Corrected S.E
[c] Lower and upper bounds (Bias-Corrected)

Supplementary Table S9. Direct and indirect effects of AL modulation by psychosocial life stressors and healthy habits mediated by perceived influence and resilience

		Direct Effects			Indirect Effects			
Modulator		Standardized (p)[a]	S.E[b]	95% CI[c]	***Mediation path***	Standardized (p)[a]	S.E[b]	95% CI[c]
Number of Events	← Perceived Influence	**0.812 (0.003)**	0.02	0.778, 0.841	← Perceived Influence ← AL	0.115 (0.078)	0.06	-0.017, 0.237
	← Resilience	-0.032 (0.374)	0.04	-0.114, 0.045	← Resilience ← AL	0.000 (0.493)	0.00	-0.006, 0.002
	← AL	0.003 (0.958)	0.07	-0.136, 0.162	← Resilience ← Perceived Influence ← AL	0.000 (0.163)	0.00	0, 0.003
Pyramid Diet Score	← Perceived Influence	-0.004 (0.776)	0.02	-0.053, 0.041	← Perceived Influence ← AL	-0.001 (0.647)	0.00	-0.012, 0.005
	← Resilience	0.032 (0.561)	0.05	-0.07, 0.116	← Resilience ← AL	0.000 (0.562)	0.00	-0.003, 0.008
	← AL	**-0.156 (0.001)**	0.04	-0.231, -0.08	← Resilience ← Perceived Influence ← AL	0.000 (0.291)	0.00	-0.003, 0.001
Cognitive Habits	← Perceived Influence	0.046 (0.07)	0.03	-0.005, 0.098	← Perceived Influence ← AL	0.006 (0.076)	0.01	-0.001, 0.023
	← Resilience	0.015 (0.753)	0.04	-0.062, 0.091	← Resilience ← AL	0.000 (0.696)	0.00	-0.002, 0.005
	← AL	-0.004 (0.969)	0.04	-0.075, 0.07	← Resilience ← Perceived Influence ← AL	0.000 (0.484)	0.00	-0.002, 0.001
Sports Habits	← Perceived Influence	-0.003 (0.903)	0.03	-0.055, 0.045	← Perceived Influence ← AL	0.000 (0.803)	0.00	-0.011, 0.007
	← Resilience	**0.148 (0.003)**	0.04	0.049, 0.229	← Resilience ← AL	0.002 (0.683)	0.01	-0.01, 0.015
	← AL	**-0.178 (0.003)**	0.04	-0.254, -0.096	← Resilience ← Perceived Influence ← AL	**-0.002 (0.027)**	0.00	-0.006, 0
Age	← Perceived Influence	-0.024 (0.267)	0.02	-0.067, 0.017				
	← Resilience	0.011 (0.757)	0.04	-0.069, 0.09				
	← AL	**0.187 (0.002)**	0.04	0.109, 0.256				
Sex	← Perceived Influence	-0.003 (0.904)	0.02	-0.048, 0.041				
	← Resilience	-0.033 (0.41)	0.04	-0.112, 0.051				
	← AL	**-0.119 (0.002)**	0.04	-0.196, -0.042				
Education	← Perceived Influence	0.004 (0.865)	0.03	-0.059, 0.067				
	← Resilience	-0.006 (0.87)	0.04	-0.093, 0.077				
	← AL	**-0.08 (0.031)**	0.04	-0.159, -0.007				
	Perceived Influence ← Resilience	**-0.088 (0.001)**	0.02	-0.137, -0.039				
	AL← Perceived Influence	0.141 (0.07)	0.08	-0.015, 0.299				
	AL ← Resilience	0.013 (0.739)	0.04	-0.065, 0.087				

S.E: Standard Error, CI: confidence interval
[a] 2-Tailed Significance (Bias-Corrected)
[b] 2-Bias-Corrected S.E
[c] Lower and upper bounds (Bias-Corrected)

Supplementary Table S10. Direct and indirect effects of AL modulation by traumatic life stressors and healthy habits mediated by perceived influence, resilience and poor sleep quality

		Direct Effects			Indirect Effects			
Modulator		Standardized (p)[a]	S.E[b]	95% CI[c]	***Mediation path***	Standardized (p)[a]	S.E[b]	95% CI[c]
Number of Events	← Perceived Influence	**0.824 (0.003)**	0.02	0.794, 0.852	← Perceived Influence ← AL	0.046 (0.435)	0.06	-0.083, 0.154
	← Resilience	0.004 (0.965)	0.04	-0.076, 0.081	← Resilience ← AL	0.000 (0.772)	0.00	-0.003, 0.006
	← AL	0.076 (0.309)	0.07	-0.062, 0.227	← Resilience ← Perceived Influence ← AL	0.000 (0.638)	0.00	-0.001, 0
					← Perceived Influence ← Sleep ← AL	**0.025 (0.001)**	0.01	0.01, 0.05
					← Resilience ← Sleep ← AL	0.000 (0.945)	0.00	-0.003, 0.003
					← Resilience ← Perceived Influence ← Sleep ← AL	0.000 (0.823)	0.00	0, 0
Pyramid Diet Score	← Perceived Influence	-0.034 (0.121)	0.02	-0.082, 0.01	← Perceived Influence ← AL	-0.002 (0.239)	0.00	-0.014, 0.002
	← Resilience	0.034 (0.514)	0.05	-0.066, 0.12	← Resilience ← AL	0.001 (0.386)	0.00	-0.002, 0.01
	← AL	**-0.176 (0.002)**	0.04	-0.248, -0.099	← Resilience ← Perceived Influence ← AL	0.000 (0.266)	0.00	-0.001, 0
					← Perceived Influence ← Sleep ← AL	-0.001 (0.082)	0.00	-0.004, 0
					← Resilience ← Sleep ← AL	-0.001 (0.404)	0.00	-0.005, 0.002
					← Resilience ← Perceived Influence ← Sleep ← AL	0.000 (0.215)	0.00	0, 0
Cognitive Habits	← Perceived Influence	0.039 (0.129)	0.03	-0.01, 0.086	← Perceived Influence ← AL	0.002 (0.287)	0.00	-0.002, 0.014
	← Resilience	0.01 (0.806)	0.04	-0.067, 0.088	← Resilience ← AL	0.000 (0.566)	0.00	-0.003, 0.007
	← AL	-0.013 (0.803)	0.04	-0.083, 0.067	← Resilience ← Perceived Influence ← AL	0.000 (0.468)	0.00	-0.001, 0
					← Perceived Influence ← Sleep ← AL	0.001 (0.062)	0.00	0, 0.004
					← Resilience ← Sleep ← AL	0.000 (0.76)	0.00	-0.003, 0.002
					← Resilience ← Perceived Influence ← Sleep ← AL	0.000 (0.571)	0.00	0, 0
Sports Habits	← Perceived Influence	-0.026 (0.31)	0.03	-0.076, 0.026	← Perceived Influence ← AL	-0.001 (0.314)	0.00	-0.011, 0.002
	← Resilience	**0.152 (0.003)**	0.04	0.061, 0.234	← Resilience ← AL	0.005 (0.318)	0.01	-0.005, 0.02
	← AL	**-0.181 (0.002)**	0.04	-0.262, -0.103	← Resilience ← Perceived Influence ← AL	0.000 (0.27)	0.00	-0.002, 0
					← Perceived Influence ← Sleep ← AL	-0.001 (0.191)	0.00	-0.003, 0
					← Resilience ← Sleep ← AL	**-0.005 (0.001)**	0.00	-0.011, -0.002
					← Resilience ← Perceived Influence ← Sleep ← AL	**0.000 (0.008)**	0.00	-0.001, 0
Age	← Perceived Influence	-0.026 (0.228)	0.02	-0.065, 0.02				
	← Resilience	0.01 (0.794)	0.04	-0.073, 0.084				
	← AL	**0.195 (0.002)**	0.04	0.121, 0.264				

Sex	← Perceived Influence	**0.065 (0.009)**	0.03	0.016, 0.113
	← Resilience	-0.035 (0.357)	0.04	-0.12, 0.045
	← AL	**-0.113 (0.003)**	0.04	-0.189, -0.035
Education	← Perceived Influence	0.004 (0.927)	0.03	-0.049, 0.05
	← Resilience	-0.006 (0.89)	0.04	-0.097, 0.077
	← AL	Restricted	-	-
	Perceived Influence ← Resilience	**-0.042 (0.036)**	0.02	-0.085, -0.004
	Poor Sleep Quality ← Perceived Influence	**0.243 (0.001)**	0.05	0.14, 0.347
	Poor Sleep Quality ← Resilience	**-0.243 (0.003)**	0.04	-0.325, -0.152
	AL← Perceived Influence	0.056 (0.447)	0.07	-0.1, 0.186
	AL ← Resilience	0.03 (0.414)	0.04	-0.04, 0.108
	AL ← Poor Sleep Quality	**0.127 (0.002)**	0.04	0.05, 0.202

S.E: Standard Error, CI: confidence interval
[a] 2-Tailed Significance (Bias-Corrected)
[b] 2-Bias-Corrected S.E
[c] Lower and upper bounds (Bias-Corrected)

Supplementary Table S11. Direct and indirect effects of AL modulation by psychosocial life stressors and healthy habits mediated by perceived influence, resilience and poor sleep quality

		Direct Effects				Indirect Effects		
Modulator		Standardized (p)[a]	S.E[b]	95% CI[c]	***Mediation path***	Standardized (p)[a]	S.E[b]	95% CI[c]
Number of Events	← Perceived Influence	**0.812 (0.003)**	0.02	0.778, 0.841	← Perceived Influence ← AL	0.094 (0.11)	0.06	-0.022, 0.212
	← Resilience	-0.032 (0.374)	0.04	-0.114, 0.045	← Resilience ← AL	-0.001 (0.282)	0.00	-0.01, 0.001
	← AL	0.003 (0.982)	0.07	-0.139, 0.156	← Resilience ← Perceived Influence ← AL	0.000 (0.176)	0.00	0, 0.002
					← Perceived Influence ← Sleep ← AL	**0.021 (0.001)**	0.01	0.008, 0.04
					← Resilience ← Sleep ← AL	0.001 (0.258)	0.00	-0.001, 0.005
					← Resilience ← Perceived Influence ← Sleep ← AL	0.000 (0.164)	0.00	0, 0
Pyramid Diet Score	← Perceived Influence	-0.004 (0.776)	0.02	-0.053, 0.041	← Perceived Influence ← AL	0.000 (0.637)	0.00	-0.009, 0.004
	← Resilience	0.032 (0.561)	0.05	-0.07, 0.116	← Resilience ← AL	0.001 (0.346)	0.00	-0.002, 0.011
	← AL	**-0.172 (0.002)**	0.04	-0.242, -0.095	← Resilience ← Perceived Influence ← AL	0.000 (0.308)	0.00	-0.002, 0
					← Perceived Influence ← Sleep ← AL	0.000 (0.745)	0.00	-0.002, 0.001
					← Resilience ← Sleep ← AL	-0.001 (0.417)	0.00	-0.005, 0.002
					← Resilience ← Perceived Influence ← Sleep ← AL	0.000 (0.328)	0.00	0, 0
Cognitive Habits	← Perceived Influence	0.046 (0.07)	0.03	-0.005, 0.098	← Perceived Influence ← AL	0.005 (0.122)	0.01	-0.001, 0.02
	← Resilience	0.015 (0.753)	0.04	-0.062, 0.091	← Resilience ← AL	0.001 (0.494)	0.00	-0.003, 0.008
	← AL	-0.017 (0.68)	0.04	-0.087, 0.062	← Resilience ← Perceived Influence ← AL	0.000 (0.474)	0.00	-0.002, 0
					← Perceived Influence ← Sleep ← AL	**0.001 (0.033)**	0.00	0, 0.004
					← Resilience ← Sleep ← AL	0.000 (0.69)	0.00	-0.003, 0.002
					← Resilience ← Perceived Influence ← Sleep ← AL	0.000 (0.539)	0.00	0, 0
Sports Habits	← Perceived Influence	-0.003 (0.903)	0.03	-0.055, 0.045	← Perceived Influence ← AL	0.000 (0.736)	0.00	-0.011, 0.006
	← Resilience	**0.148 (0.003)**	0.04	0.049, 0.229	← Resilience ← AL	0.006 (0.192)	0.01	-0.004, 0.023
	← AL	**-0.174 (0.003)**	0.04	-0.253, -0.094	← Resilience ← Perceived Influence ← AL	-0.001 (0.051)	0.00	-0.005, 0
					← Perceived Influence ← Sleep ← AL	0.000 (0.899)	0.00	-0.002, 0.001
					← Resilience ← Sleep ← AL	**-0.004 (0.001)**	0.00	-0.01, -0.002
					← Resilience ← Perceived Influence ← Sleep ← AL	**0.000 (0.000)**	0.00	-0.001, 0
Age	← Perceived Influence	-0.024 (0.267)	0.02	-0.067, 0.017				
	← Resilience	0.011 (0.757)	0.04	-0.069, 0.09				
	← AL	**0.194 (0.002)**	0.04	0.119, 0.262				

Sex	← Perceived Influence	-0.003 (0.904)	0.02	-0.048, 0.041
	← Resilience	-0.033 (0.41)	0.04	-0.112, 0.051
	← AL	**-0.122 (0.002)**	0.04	-0.194, -0.043
Education	← Perceived Influence	0.004 (0.865)	0.03	-0.059, 0.067
	← Resilience	-0.006 (0.87)	0.04	-0.093, 0.077
	← AL	Restricted	-	-
	Perceived Influence ← Resilience	**-0.088 (0.001)**	0.02	-0.137, -0.039
	Poor Sleep Quality ← Perceived Influence	**0.199 (0.001)**	0.04	0.121, 0.288
	Poor Sleep Quality ← Resilience	**-0.229 (0.002)**	0.04	-0.315, -0.143
	AL← Perceived Influence	0.116 (0.116)	0.08	-0.03, 0.26
	AL ← Resilience	0.042 (0.283)	0.04	-0.033, 0.116
	AL ←Poor Sleep Quality	**0.13 (0.001)**	0.04	0.056, 0.209

S.E: Standard Error, CI: confidence interval
[a] 2-Tailed Significance (Bias-Corrected)
[b] 2-Bias-Corrected S.E
[c] Lower and upper bounds (Bias-Corrected)
